# Supplementary material for: Posterior scleral birefringence measured by triple-input polarization-sensitive imaging as a biomarker of myopia progression
Source: Nat Biomed Eng. 2023 Jun 26;7(8):986–1000. doi: 10.1038/s41551-023-01062-w (PMC10427432; doi:10.1038/s41551-023-01062-w)
Supplement: Supplementary file 1 — Supplementary discussion, methods, figures, tables and references. [file 41551_2023_1062_MOESM1_ESM.pdf]

nature biomedical engineering

Article

https://doi.org/10.1038/s41551-023-01062-w

Posterior scleral birefringence measured by triple-input polarization-sensitive imaging as a biomarker of myopia progression

In the format provided by the authors and unedited

Supplementary Information

Supplementary Discussion 1 | Edge-artifacts in local retardance reconstruction
Supplementary Discussion 2 | Collagen fiber diameter and scleral birefringence
Supplementary Discussion 3 | Image quality criteria and penetration limitation

Supplementary Method 1 | Reconstruction of a pure Mueller matrix in TRIPS-OCT
Supplementary Method 2 | Dual-input, Jones matrix, and single-input birefringence reconstruction methods
Supplementary Method 3 | Generation of mutually orthogonal triple polarization states
Supplementary Method 4 | Recovery of the reciprocal constraint for depth cumulative Mueller matrices
Supplementary Method 5 | Spectral binning to remove wavelength-dependent polarization variation
Supplementary Method 6 | Compensation of corneal retardance and diattenuation
Supplementary Method 7 | Validation of depth-resolved optic axis measurement using a phantom
Supplementary Method 8 | Enface birefringence image projection of guinea pigs and humans
Supplementary Method 9 | Human retina imaging interface

Supplementary Data 1 | In-vivo TRIPS-OCT images of a guinea pig at 16-week-old before TEM analysis
Supplementary Data 2 | Longitudinal observations of scleral birefringence development during refraction development in guinea pigs
Supplementary Data 3 | Scleral birefringence and refractive error in guinea pigs from ages of 2-8 weeks
Supplementary Data 4 | Location dependence of scleral birefringence and myopia status in the emmetropia and low myopia group
Supplementary Data 5 | Correlation between biometrics of the human eyes in the emmetropia and low myopia group
Supplementary Data 6 | Grouping of eyes and subjects and characteristics

Supplementary Discussion 1: Edge-artifacts in local retardance reconstruction

The edge-artifact is a fundamental noise source of polarization sensitive detection in PS-OCT. The polarization states of light backscattered from the sample are recorded by the relative amplitude and phase between two detection channels principally configured at the two orthogonal outputs of a polarized beam splitter. Considering the axial scan of a particle with the size smaller than the axial resolution, ideally, the point-spread functions (PSFs) of the particle in the two detection channels should perfectly align. However, the birefringence in the superficial layers of the sample (including the cornea and optical components in the instrument), induces polarization mode dispersion and results in a differential group delay between the two principal polarization modes. Therefore, a shift in the PFSs is created and leads to a relative amplitude difference along the ranging depth which does not truly reflect the polarization states but is an artifact.

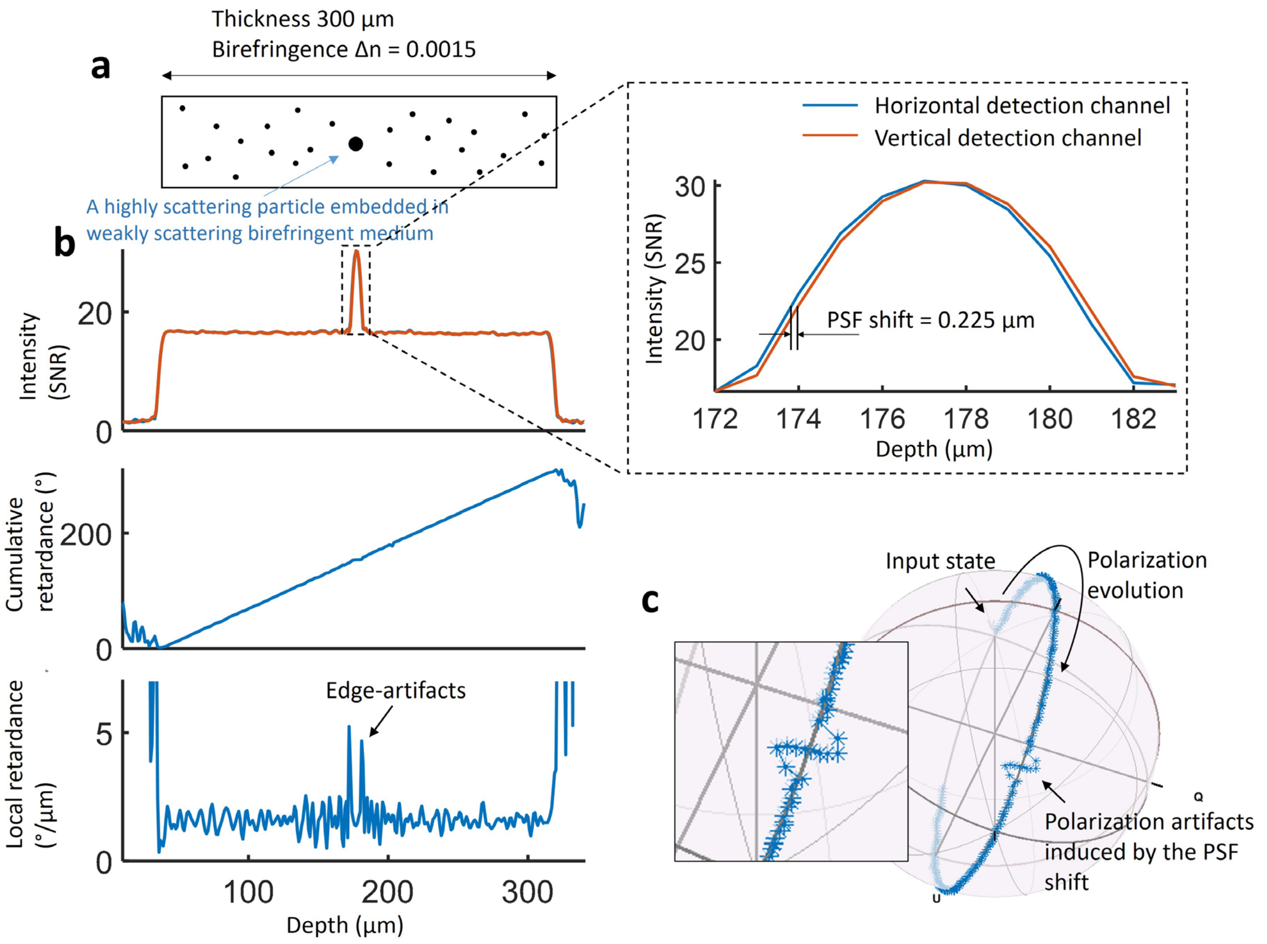

Supplementary Discussion Fig. 1 | Simulated edge-dependent artifacts in local retardance reconstruction. a, Sample structure used in the PSOCT simulation. **b**, Reconstructed sample profiles of intensity, cumulative retardance and local retardance. **c**, The trace of polarization state evolution of light backscattered from the sample.

To illustrate the edge-artifacts, we simulated the axial scan of a particle embedded in a weakly-scattering, birefringent medium and reconstructed the cumulative and local retardance of the sample. Specifically, we simulated the axial scans of a 300-µm-thick sample made of a weakly scattering birefringent medium (Δn = 0.0015) with one highly scattering particle embedded in the middle of the medium (**Supplementary Discussion Fig. 1a**). In this simulation, circularly polarized light was used as the input state. The optic axis of the sample was set as 0° (aligned with the horizontal channel of the detection system). OCT scans were simulated by generating the fringes in the wavenumber domain of the individual scatterers and then transforming the summed fringes into the depth domain using Fourier transformation. To suppress the speckle noise, we averaged 1000 simulated A-lines with random speckle patterns and obtained the depth profile and the cumulative and local retardance of the sample (**Supplementary Discussion Fig 1b**). We assume that the imaging system does not have polarization mode dispersion; therefore, spectral binning is not performed in the simulation. In the profile of the highly scattering particle, a shift in PSFs between the two polarization sensitive detection channels can be seen. In this simulation, the length of shift was determined by the

cumulative retardance of the superficial layer (Δn x 150 μm). Cumulative retardance was calculated as the rotation angle from the input state to the measured polarization states on the Poincare sphere and local retardance was calculated as the local rotation angle of the Stokes vectors within 1-μm section along the depth. The theoretical evolution trace of the polarization state should locate in the UV-plane on the Poincare sphere; but due to the PSF shift, measured polarization states deviate from the theoretical trace, creating artifacts in reconstructed retardance. The derivative nature of the local retardance profile makes it more sensitive to these artifacts, and thus, the edge-artifacts are more obvious in the local retardance profile.

These edge-artifacts are also related to the absolute polarization state of the light, the eigenstates of the superficial sample, and the direction of the detection channels. These artifacts are more obvious when the PSFs of the two channels are comparable in amplitude. In particular, for a specific polarization state where the backscattered light is directed to only one detection channel, no edge-artifacts would exist in the reconstruction.

These edge-artifacts cannot be removed by kernel averaging as they are associated with intensity variation, which is the information carrier of the sample structure. Interestingly, these artifacts, induced by unwanted amplitude differences of the shifted PSFs in two polarization channels, are physically behaving like sample diattenuation, and therefore, the artifacts can be suppressed by removing the sample diattenuation component from the Mueller matrix using polar decomposition when reconstructing the local birefringence properties.

By further studying the noise sources in different birefringence reconstruction methods (**Ext. Data Fig. 3**), we find that the birefringence sensitivity is fundamentally limited by the intensity SNR in all PS-OCT reconstruction methods. With a sufficient intensity signal (SNR > 10 dB), birefringence noise is dominated by the edge-artifacts if pure retardance is assumed in the reconstruction.

Supplementary Discussion 2: Collagen fiber diameter and scleral birefringence

The reasons for scleral birefringence increasing with collagen fiber diameter may include (1) increased form birefringence because of a larger volume fraction of the fibrillar collagen; (2) increased intrinsic birefringence due to larger collagen content; and (3) thickening of collagen lamellas in the sclera.

Scleral form birefringence in a localized region can be crudely modelled by parallel collagen cylinders embedded in an extrafibrillar matrix. Revisiting Wiener's theory[1] for birefringence of a diluted solution of cylinders, for a dielectric solvent with a refractive index n_1 and volume fraction f_1 consisting well-separated, parallelly-aligned cylinders with a refractive index n_2 and volume fraction f_2, the refractive indices parallel and perpendicular to the cylinder axes, n_e and n_o, are expressed by

$$n_e^2 = n_1^2 f_1 + n_2^2 f_2,$$

$$n_o^2 = n_1^2 + \frac{(n_2^2 - n_1^2) f_2}{1 + \frac{\gamma f_1}{2}},$$

where $\gamma = (n_2^2 - n_1^2)/n_1^2$. We used the parameters in a recent report[2] using the refractive index of fibrillar collagen $n_2 = 1.504$, and extrafibrillar matrix $n_1 = 1.345$ to model the sclera. From this model, scleral birefringence is increasing with the fibrillar collagen volume fraction f_2 in the range of 0 to 0.5 and reaches a maximum when $f_2 = 0.5$ (**Supplementary Discussion Fig. 2a**).

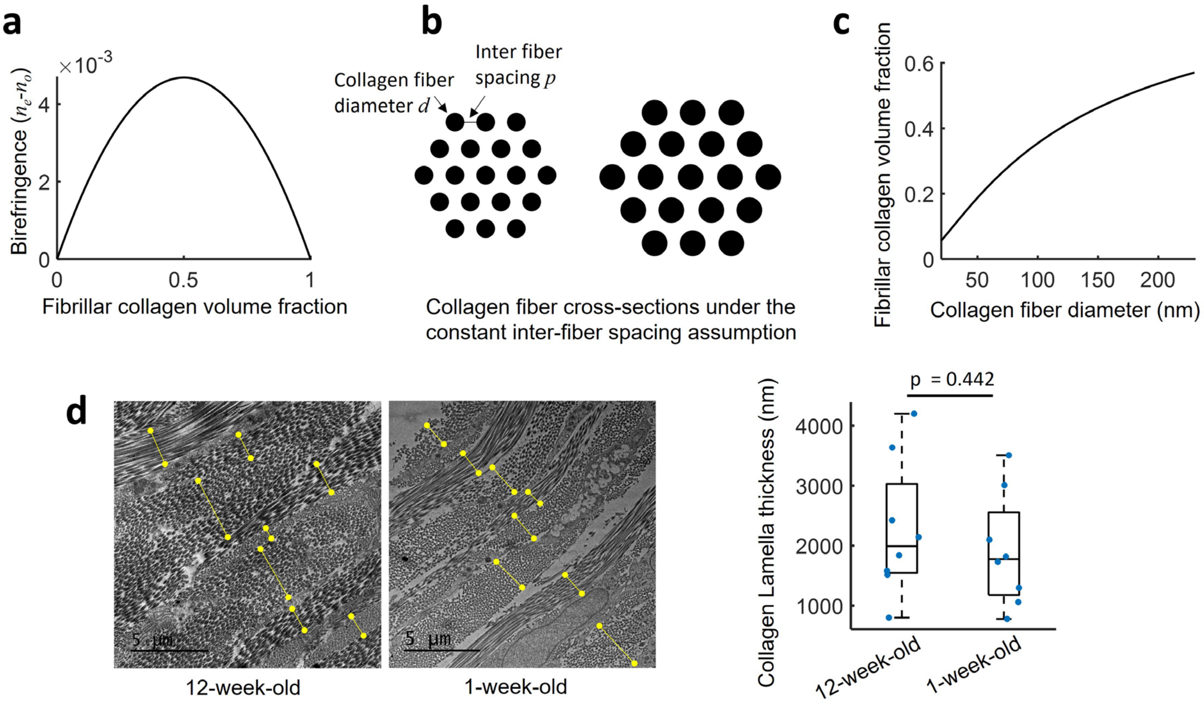

Supplementary Discussion Fig. 2 | Scleral birefringence increases with the enlargement of collagen fiber. a, Form birefringence model based on parallel collagen cylinders embedded in an extrafibrillar matrix. **b**, Geometric model of sclera under constant inter-fiber spacing. **c**, Fibrillar collagen volume fraction as a function of fiber diameter using the geometric model. **d**, Thickening of collagen lamellas during the development of sclera. Dots represent n = 8 individual lamellas from 1 sclera sample for each group, central line indicates median, box shows interquartile range and whiskers show range. The *p* value is calculated by two-sided Wilcoxon rank sum test.

It has been reported that the human sclera contains approximately 50% collagen by weight[3], with type I fibrillar collagen being the main sub-type. The diameter of collagen fibers varies between 20 to 230 nm and the spacing between collagen fibers is highly irregular and was reported to have an average length of 285 nm (fiber center-to-center, corresponding to a minimum of around 60 nm edge-to-edge) in human sclera[4]. Electron microscopy may not be a proper technique to estimate the volume fraction due to the tissue shrinkage in the preparation process[5]. Using a crude geometric model to study the relationship between the collagen fiber diameter and its

volume fraction, we assume the average spacing between fibers (edge-to-edge) to be a constant. This assumption is based on the understanding of the necessary space for the microstructures of the matrix including decorin, biglycan, and glycosaminoglycan forming cross-linking bridges between adjacent fibers. In this geometric model, the fibers are naturally arranged in a hexagonal pattern (**Supplementary Discussion Fig. 2b**). From this model, with the edge-to-edge inter-fiber spacing set as 60 nm, as the fiber diameter increases from 20 to 230 nm, the fibrillar collagen volume fraction increases from 0.057 to 0.57 (**Supplementary Discussion Fig. 2c**).

We attempted to use this model to qualitatively explain the increased birefringence associated with the increased fiber diameter, specifically, the relationship between the collagen fiber diameter and its volume fraction. The limitations of this model include that, first, the collagen fiber diameter is highly variable even within a single lamella; second, the spacing between the fibers is highly irregular; third, the collagen fiber arrangement in a lamella is not parallel but curved and wavy; fourth, Wiener's theory is only accurate in diluted solution where $f_2 \ll 1$. Considering all of these, the form birefringence model of the sclera is crude and limited but provides an explanation of the increased form birefringence with larger collagen fiber diameter. Further investigations are needed to establish an accurate model for the form birefringence in sclera.

In addition to the form birefringence, it has been reported that collagen is highly intrinsically birefringent due to the bonds of protein molecules[6]. The intrinsic birefringence is proportional to the collagen content in the sclera and may also contribute to the increase of total birefringence with a larger collagen fiber diameter.

The collagen fibers are similarly arranged within each lamella, therefore, thickening of lamellas are correlated with the increase of observed birefringence due to more aligned fibers within a resolution volume. In the scleral tissue of guinea pigs from ages of 1 and 12 weeks, we observed that the thickness of lamella was increasing with age (**Supplementary Discussion Fig. 2d**) although statical significance were not achieved. It has been reported that the growth of sclera in tree shrews is associated with a positive skewing of the distribution of the collagen fiber diameter from a relatively normal distribution profile at birth[7], consistent with our observations in guinea pigs. The thickening of the lamella is highly likely a result of the enlargement of collagen fibers in the lamella and is supposed to be one of the reasons of the increase of scleral birefringence.

Putting everything together, a larger average collagen fiber diameter in the sclera leads to increased PSB in TRIPS-OCT images.

Supplementary Discussion 3: Image quality criteria and penetration limitation

The workflow of image quality check for TRIPS-OCT human data included two steps. First, by inspecting the volume scan of the retina, we excluded 50 datasets with suboptimal depth or lateral positioning, or inaccurate pupil alignment (**Supplementary Discussion Fig. 3a**). Second, on the remaining datasets, the choroidal-scleral interface was manually labelled, and the average scleral intensity signal-to-noise ratio (SNR) was estimated from a 100-um slab below the manually labelled choroidal-sclera interface. Datasets with an average scleral intensity SNR lower than 4.6 dB were excluded from the analysis.

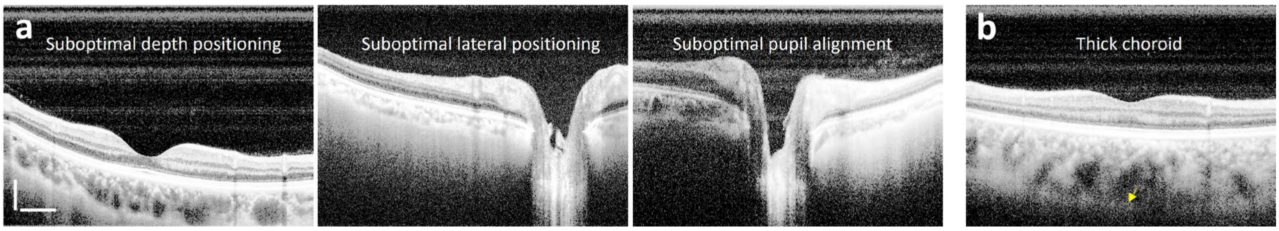

Supplementary Discussion Fig. 3 | Example datasets excluded in the analysis. a, Cross-sectional images in datasets excluded by the reason of suboptimal positioning. **b**, Cross-sectional images in datasets excluded by the reason of insufficient intensity signal from the sclera. The yellow arrow in **b** indicate the choroidal-scleral interface. Scale bars, vertical: 300 μm, horizontal: 1 mm.

A relatively large portion (50 of 180) of the data was rejected by inappropriate alignment of the scanning head. As a first-in-human study, comparing to a commercial device, the TRIPS-OCT lacks a fundus preview, multiple location B-scan previews, a motor-driven reference mirror, and a retina tracker. Without these alignment-aiding techniques, it is relatively difficult to align the scanning head to the right position. Repetitive imaging was limited because TRIPS-OCT is classified as a dose-limited Group I system according to the American National Standard for Ophthalmics[8], meaning that no potential light hazard is allowed to arise from the procedure, which limits the total laser exposure time on patients within 24 hours. In our study, considering the patients may already undergo eye examinations in the same visit by other two OCT modalities in our research clinic, to minimize the laser dose exposed on the patients on a single day, our protocol did not allow for repetitive scans more than two times in one patient during one visit. For the abovementioned reasons, the rejection rate was high in this study due to inappropriate positioning. However, we believe that this issue can be minimized by the further engineering development of the alignment system.

TRIPS-OCT penetration is fundamentally limited by the intensity SNR. In this study, we excluded 25 (of 180) datasets due to insufficient intensity signal from the sclera. We found that thick choroid (roughly > 450 μm) (**Supplementary Discussion Fig. 3b**) was one of the main reasons blocking the visibility of the sclera. To overcome this limitation, techniques to further enhance the imaging sensitivity of TRIPS-OCT are still needed.

Supplementary Method 1: Reconstruction of a pure Mueller matrix in TRIPS-OCT

In our TRIPS-OCT system, the detected interferometric fringes undergo 3 steps to reconstruct the cumulative Mueller matrix, including (1) filtering in Stokes domain, (2) minimizing the depolarization effect, and last, (3) solving a pure Mueller matrix.

1. Filtering in Stokes domain

Considering the fringes $F_{Hn}(x,k)$ and $F_{Vn}(x,k)$ of one Bscan from two polarization diverse detection channels, complex tomograms $E_{Hn}(x,z)$ and $E_{Vn}(x,z)$ can be obtained by Fourier transformation of the fringes from k-domain to z-domain, where x represent the lateral scanning position and z represents the depth. n represents the index of repetitive scans in one location and $n = 1,2,3$. Stokes vectors of the image pixels can be reconstructed by

$$\mathbf{S}_n(x,z) = \begin{bmatrix} |E_{Hn}(x,z)|^2 + |E_{Vn}(x,z)|^2 \\ |E_{Hn}(x,z)|^2 - |E_{Vn}(x,z)|^2 \\ 2Re(E_{Hn}(x,z)E_{Vn}^*(x,z)) \\ -2Im(E_{Hn}(x,z)E_{Vn}^*(x,z)) \end{bmatrix}. \dots (1)$$

* represents the complex conjugate. To suppress noise, spatial filtering was performed on Stokes vectors by convolving the components of Stokes vectors with a filter kernel $\mathbf{k}$, where $\tilde{\mathbf{S}}_{\mathrm{n}}(x,z) = \mathbf{S}_n(x,z)\otimes\mathbf{k}$.

In the TRIPS-OCT system, three sets of Stokes vectors are acquired, reconstructed, and filtered, noted as $\tilde{\mathbf{S}}_1(x,z), \tilde{\mathbf{S}}_2(x,z), \tilde{\mathbf{S}}_3(x,z)$. Without loss of generality, the input probing light with three orthogonal input states before going through the system and sample can be assembled into a probing matrix $\mathbf{s}$.

$$\mathbf{s} = \begin{bmatrix} 1 & 1 & 1 \\ 1 & 0 & 0 \\ 0 & 1 & 0 \\ 0 & 0 & 1 \end{bmatrix}. \dots (2)$$

The reconstructed Stokes vectors corresponding to the three input states can be assembled into a 4x3 detection matrix $\boldsymbol{\mu}(x,z) = [\tilde{\mathbf{S}}_1(x,z) \quad \tilde{\mathbf{S}}_2(x,z) \quad \tilde{\mathbf{S}}_3(x,z)]$. Using a Mueller matrix to describe the entire optical system and sample from the probing matrix to the detection,

$$\boldsymbol{\mu}(x,z) = \mathbf{M}(x,z)\mathbf{s}. \dots (3)$$

$\mathbf{M}(x,z)$ is the unknown Mueller matrix. To simplify the notation, we omit the pixel coordinate (x,z) in the following steps, as the operations are applied indiscriminately to every pixel in the image.

2. Minimizing the depolarization effect

A general full Mueller matrix can be decomposed as

$$\mathbf{M} = \mathbf{DL}. \dots (4)$$

where $\mathbf{L}$ is a pure Mueller matrix, $\mathbf{D}$ is a general depolarization matrix. A pure Mueller matrix, or Jones-derived Mueller matrix, belongs to the subset of physically admissible Mueller matrices describing a non-depolarizing medium.

In an OCT system, the detected interferometric signal arises only from the component of the backscattered light that is coherently mixed with the reference light. An individual measurement is by definition fully polarized and non-depolarized; therefore, the entire optical system and sample can be described by a pure Mueller matrix. In this ideal case, $\mathbf{D}$ should be an identity matrix and $\mathbf{M}$ should be in the form of $\mathbf{L}$. However, spatial filtering to the Stokes vectors is equivalent to introducing depolarization[9,10]. The induced depolarization, either from noise or truly depolarizing structures, undermines the pure Mueller form of $\mathbf{M}$.

Full determination of the underlying Muller matrix $\mathbf{M}$ would require the measurement its 16 (constrained) parameters. In TRIPS-OCT, an approximate solution is found by fitting a pure Mueller matrix $\mathbf{L}$ to the available measurements $\boldsymbol{\mu}$. Specifically, we attempt to find the $\mathbf{L}$ that results in observed polarization states $\boldsymbol{\mu}' = \mathbf{Ls}$, which align with the polarized component of the actually measured states.

To this end, we first polarize the observed states $\boldsymbol{\mu}$ by replacing their filtered i components with the Euclidean norms of the q, u, v components by $\tilde{\imath}_n = \sqrt{\tilde{q}_n^2 + \tilde{u}_n^2 + \tilde{v}_n^2}$, where $\tilde{\mathbf{S}}_\mathrm{n} = [\tilde{\imath}_n \quad \tilde{q}_n \quad \tilde{u}_n \quad \tilde{v}_n]^\mathrm{T}$ to obtain $\tilde{\boldsymbol{\mu}}$ by

$$\tilde{\boldsymbol{\mu}} = \begin{bmatrix} \tilde{\imath}_1 & \tilde{\imath}_2 & \tilde{\imath}_3 \\ \tilde{q}_1 & \tilde{q}_2 & \tilde{q}_3 \\ \tilde{u}_1 & \tilde{u}_2 & \tilde{u}_3 \\ \tilde{v}_1 & \tilde{v}_2 & \tilde{v}_3 \end{bmatrix} . \ldots (5)$$

Second, a necessary condition of pure Mueller matrix[11] is that $\mathbf{L}^\mathrm{T}\mathbf{G}\mathbf{L} = c^2\mathbf{G}$, where $\mathbf{G}$ is a diagonal matrix defined as $\mathrm{diag}(1, -1, -1, -1)$. c is a scalar. Therefore, $\boldsymbol{\mu}'$ should satisfy that

$$\boldsymbol{\mu}'^\mathrm{T}\mathbf{G}\boldsymbol{\mu}' = \mathbf{s}^\mathrm{T}\mathbf{L}^\mathrm{T}\mathbf{G}\mathbf{L}\mathbf{s} = c^2\mathbf{s}^\mathrm{T}\mathbf{G}\mathbf{s} = c^2\mathbf{H}. \ldots (6)$$

where $\mathbf{H} = \begin{bmatrix} 0 & 1 & 1 \\ 1 & 0 & 1 \\ 1 & 1 & 0 \end{bmatrix}$. For the observed and polarized polarization states $\tilde{\boldsymbol{\mu}}$ to meet this condition, they have to be rebalanced with a 3×3 diagonal matrix $\mathbf{A} = \mathrm{diag}(a_1, a_2, a_3)$ to obtain $\boldsymbol{\mu}' = \tilde{\mathbf{u}}\mathbf{A}$. Plugging this into eq. (6) gives

$$\mathbf{A}^\mathrm{T}\tilde{\mathbf{u}}^\mathrm{T}\mathbf{G}\tilde{\mathbf{u}}\mathbf{A} = c^2\mathbf{H}. \ldots (7)$$

Although this matrix equation contains 9 sub-equations, we find that the number of constraints is 4, which allows for exact solutions of c, a_1, a_2, a_3:

$$c = \frac{1}{3}(\tilde{\imath}_1\tilde{\imath}_2 + \tilde{\imath}_1\tilde{\imath}_3 + \tilde{\imath}_2\tilde{\imath}_3 - \tilde{q}_1\tilde{q}_2 - \tilde{q}_1\tilde{q}_3 - \tilde{q}_2\tilde{q}_3 - \tilde{u}_1\tilde{u}_2 - \tilde{u}_1\tilde{u}_3 - \tilde{u}_2\tilde{u}_3 - \tilde{v}_1\tilde{v}_2 - \tilde{v}_1\tilde{v}_3 - \tilde{v}_2\tilde{v}_3). \ldots (8)$$

$$a_1 = c\frac{(\tilde{\imath}_1\tilde{\imath}_2 - \tilde{q}_1\tilde{q}_2 - \tilde{u}_1\tilde{u}_2 - \tilde{v}_1\tilde{v}_2)(\tilde{\imath}_1\tilde{\imath}_3 - \tilde{q}_1\tilde{q}_3 - \tilde{u}_1\tilde{u}_3 - \tilde{v}_1\tilde{v}_3)}{\tilde{\imath}_2\tilde{\imath}_3 - \tilde{q}_2\tilde{q}_3 - \tilde{u}_2\tilde{u}_3 - \tilde{v}_2\tilde{v}_3}. \ldots (9)$$

$$a_2 = c\frac{(\tilde{\imath}_1\tilde{\imath}_2 - \tilde{q}_1\tilde{q}_2 - \tilde{u}_1\tilde{u}_2 - \tilde{v}_1\tilde{v}_2)(\tilde{\imath}_2\tilde{\imath}_3 - \tilde{q}_2\tilde{q}_3 - \tilde{u}_2\tilde{u}_3 - \tilde{v}_2\tilde{v}_3)}{\tilde{\imath}_1\tilde{\imath}_3 - \tilde{q}_1\tilde{q}_3 - \tilde{u}_1\tilde{u}_3 - \tilde{v}_1\tilde{v}_3}. \ldots (10)$$

$$a_3 = c\frac{(\tilde{\imath}_2\tilde{\imath}_3 - \tilde{q}_2\tilde{q}_3 - \tilde{u}_2\tilde{u}_3 - \tilde{v}_2\tilde{v}_3)(\tilde{\imath}_1\tilde{\imath}_3 - \tilde{q}_1\tilde{q}_3 - \tilde{u}_1\tilde{u}_3 - \tilde{v}_1\tilde{v}_3)}{\tilde{\imath}_1\tilde{\imath}_2 - \tilde{q}_1\tilde{q}_2 - \tilde{u}_1\tilde{u}_2 - \tilde{v}_1\tilde{v}_2}. \ldots (11)$$

$\tilde{\imath}_n, \tilde{q}_n, \tilde{u}_n, \tilde{v}_n$ are the elements of $\tilde{\boldsymbol{\mu}}$. Thereby, $\boldsymbol{\mu}'$ is reconstructed and the scaling factor c is solved.

3. Solving the pure Mueller matrix

A pure Mueller matrix $\mathbf{L}$ can be modelled as a combination of a retarder $\mathbf{L}_R$ and a diattenuator $\mathbf{L}_D$, both with unitary determinant.

$$\mathbf{L} = c\mathbf{L}_R\mathbf{L}_D. \ldots (12)$$

$\mathbf{L}_R$ is a general retarder in the form of $\mathbf{L}_R = \begin{bmatrix} 1 & 0 \\ 0 & \mathbf{m}_R \end{bmatrix}$, constrained by conditions of $\mathbf{m}_R \in \mathrm{SO}(3)$, and $\det(\mathbf{L}_R) = 1$; $\mathbf{L}_D$ is a general diattenuation in the form of $\mathbf{L}_D = \begin{bmatrix} \cosh(a) & \sinh(a) \cdot \mathbf{a}^\mathrm{T} \\ \sinh(a) \cdot \mathbf{a} & \mathbf{a} \cdot \mathbf{a}^\mathrm{T}(\cosh(a) - 1) + \mathbf{I} \end{bmatrix}$, equally with $\det(\mathbf{L}_D) = 1$ and $|\mathbf{a}|_2 = 1$, where **I** represents the 3-by-3 identity matrix, and a is a scalar. c is the overall scaler solved in eq (8).

To solve $\mathbf{L}$ from $\boldsymbol{\mu}'$, we have that

$$\boldsymbol{\mu}' = \mathbf{L}\mathbf{s} = c \cdot \begin{bmatrix} \cosh(a) + \sinh(a) \cdot \mathbf{a}^T \\ \mathbf{m}_R \cdot [\sinh(a) \cdot \mathbf{a} + \mathbf{a} \cdot \mathbf{a}^T(\cosh(a) - 1) + \mathbf{I}] \end{bmatrix} . \ldots (13)$$

To simplify the expression, we denote $\boldsymbol{\mu}' = \begin{bmatrix} \mathbf{i}' \\ \mathbf{m} \end{bmatrix}$, which is the solved fitted detection matrix. By observing eq. (13), we find that

$$\cosh(a) = \frac{\sum \mathbf{i}' - \det(\mathbf{m})}{2} . \ldots (14)$$

Hence, the vector $\mathbf{a}$ is solved by

$$\mathbf{a}^{\mathrm{T}} = \frac{\mathbf{i}' - \cosh(a)}{\sinh(a)} . \ldots (15)$$

$\mathbf{m}_R$ is then solved by

$$\mathbf{m}_R = \mathbf{m}\mathbf{m}_D^{-1}, \ldots (16)$$

where $\mathbf{m}_D$ is defined as

$$\mathbf{m}_D = [\sinh(a)\,\mathbf{a} \quad \sinh(a)\,\mathbf{a} \quad \sinh(a)\,\mathbf{a}] + \mathbf{a}\mathbf{a}^{\mathrm{T}}(\cosh(a) - 1) + \mathbf{I}. \ldots (17)$$

To conclude, the 4x4 pure Mueller matrix $\mathbf{L}$ is reconstructed by

$$\mathbf{L} = \mathbf{L}_R \cdot \mathbf{L}_D = \mathrm{c}\begin{bmatrix} 1 & 0 \\ 0 & \mathbf{m}_R \end{bmatrix}\begin{bmatrix} \cosh(a) & \sinh(a) \cdot \mathbf{a}^{\mathrm{T}} \\ \sinh(a) \cdot \mathbf{a} & \mathbf{a} \cdot \mathbf{a}^{\mathrm{T}}(\cosh(a) - 1) + \mathbf{I} \end{bmatrix} . \ldots (18)$$

Supplementary Method 2: Dual-input, Jones matrix, and single-input birefringence reconstruction methods

We numerically simulate different PS-OCT reconstruction methods that are adopted from previous reports[12–14]. The procedures are summarised in **Supplementary Method Fig. 1**.

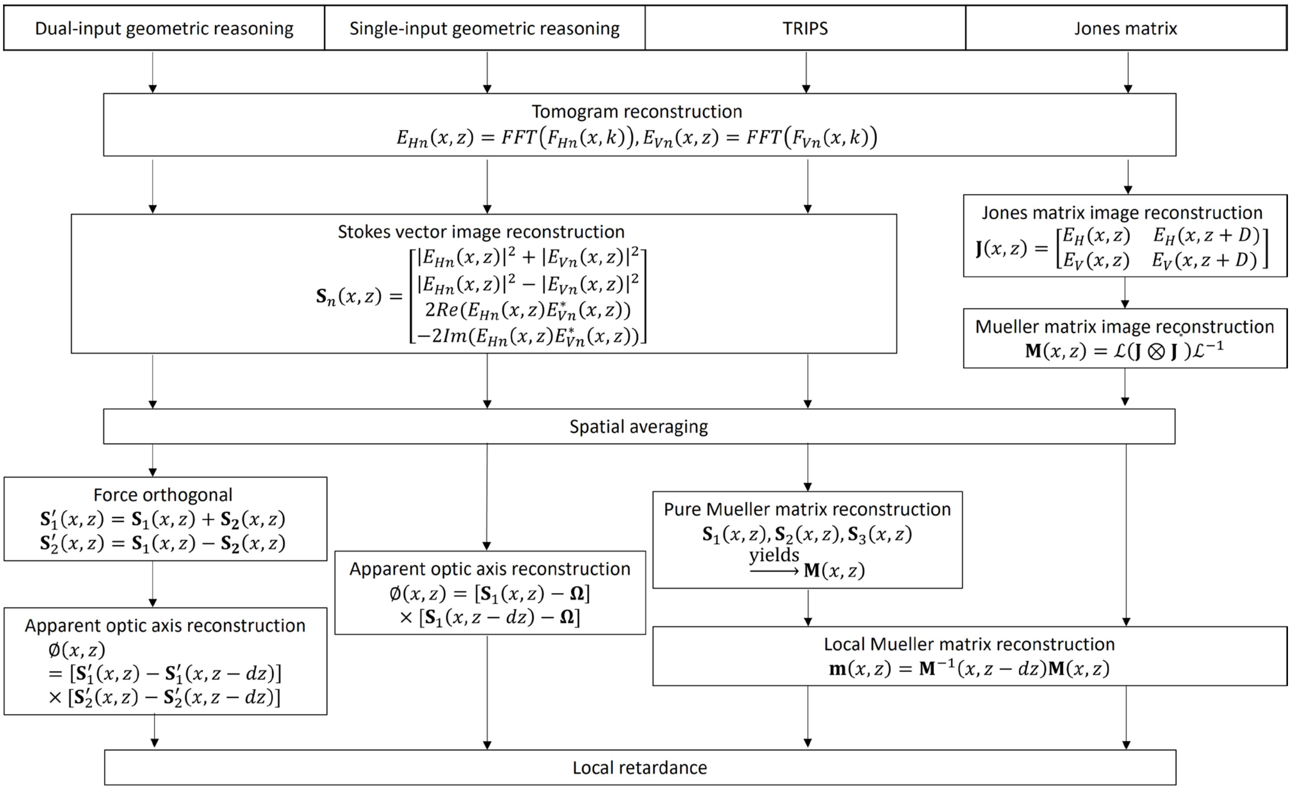

Supplementary Method Fig. 1 | Diagram of local retardance reconstruction methods including dual-input geometric reasoning, single-input geometric reasoning, TRIPS proposed in this study, and Jones matrix method used in a depth-encoding system.

$F_{Hn}(x,k)$ and $F_{Vn}(x,k)$ represent the fringes detected from two polarization diverse channels in a B-scan. x is the lateral position, k is wavenumber, and d is depth position. n represents the index of repetitive scans in a sample location, where $n=1$ in a single-input PS-OCT, $n=1,2$ in a dual-input PS-OCT, and $n=1,2,3$ in a TRIPS-OCT. In a depth-encoding PS-OCT, $n=1$ and D represents the delay length between the two orthogonal polarization imaging ranges. FFT represents Fourier transform. * represents the conjugate. $\otimes$ represents the Kronecker product. $\mathcal{L}=\begin{bmatrix}1&0&0&1\\1&0&0&-1\\0&1&1&0\\0&i&-i&0\end{bmatrix}$. $\mathbf{\Omega}$ is the constraint of the polarization state evolution[13].

In the geometric reasoning methods, the local polarization states and apparent optic axis are projected to the surface of a Poincare sphere. Over a local depth dz, the polarization state is rotating about the apparent optic axis, and the local retardance is the rotation angle within the dz range. In the TRIPS and Jones matrix methods, the differential Mueller matrix $\mathbf{a}(x,z)$ can be calculated by matrix logarithm of the local Mueller matrix $\mathbf{m}(x,z)$, where $\mathbf{a}(x,z)=\ln[\mathbf{m}(x,z)]$. The non-depolarization component is evaluated by calculating the G-antisymmetric part of $\mathbf{a}(x,z)$, where $\mathbf{b}(x,z)=[\mathbf{a}(x,z)-\mathbf{G}\mathbf{a}^{\mathrm{T}}(x,z)\mathbf{G}]/2$, and $\mathbf{G}=$ diag(1,-1,-1,-1). Local retardance is retrieved from the norm of the vector $[b_{3,4}\quad b_{4,2}\quad b_{2,3}]$, where $b_{i,j}$ is i,j entry of $\mathbf{b}$.

Supplementary Method 3: Generation of mutually orthogonal triple polarization states

Two linear polarizers sandwiching an electric-optic modulator (EOM), also known as a Pockels cell, is a common optical configuration to modulate the amplitude of light. Here, we demonstrate that 3 mutually orthogonal triple polarization states can be generated through a linear polarizer and an EOM, as shown in **Supplementary Method Fig. 2a**. The trace of polarization state evolution on the Poincaré sphere is an arc when applying a voltage to the EOM to change its retardance (**Supplementary Method Figs. 2b,c**). The radius of the arc, R, on a unit sphere is determined by the angle, α, between the orientation of the polarizer and the fast optic axis of the EOM, where $R = \sin(2\alpha)$. On a unit sphere, an arc to evolve through 3 orthogonal points has a radius of $\sqrt{6}/3$. Hence, α is solved as 27.37°. Retardance values of -120°, 0°, and -120° in such a configuration produce 3 mutually orthogonal polarization states on the Poincaré sphere (**Supplementary Method Fig. 2d**). In a fiber-based system, the absolute polarization states are randomized, yet the relative positions on the Poincaré sphere are determined for these 3 polarization states.

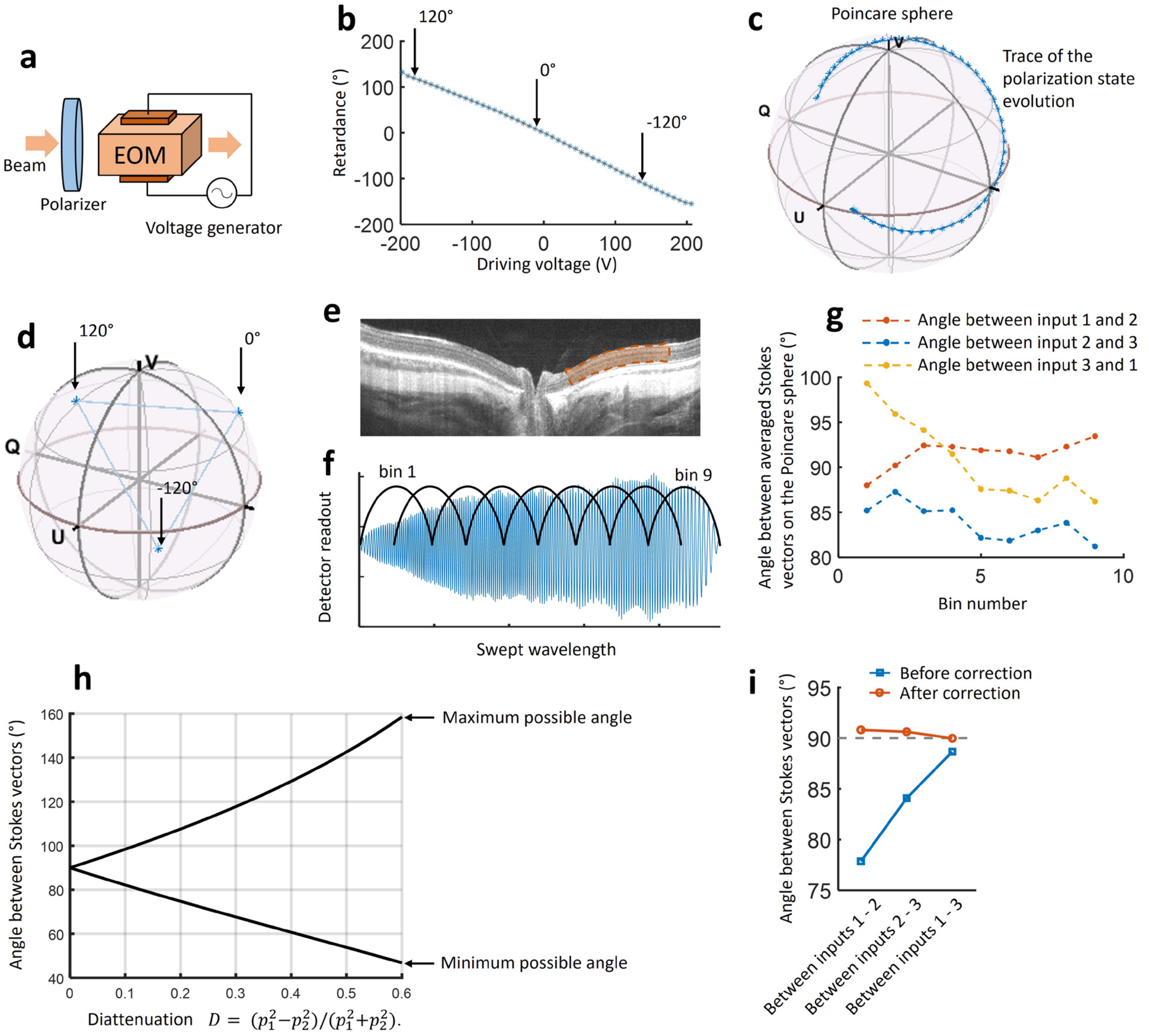

Supplementary Method Fig. 2 | Generation of mutually orthogonal triple polarization states. a, Optical configuration of the triple state modulator. The angle between the linear polarizer preceding the EOM and the optic axis of the modulator is set to 27.37°. **b**, Driving voltage against the modulated retardance. The EOM approximately linearly responds to the driving voltage. **c**, Trace of the polarization evolution on the Poincaré sphere with a linearly swept driving voltage. **d**, Driving the EOM with a 3-stage step waveform with voltages corresponding to retardances of 120°, 0°, and -120° produces 3 polarization stages that are mutually orthogonal. **e**, An example B-scan image of a guinea pig retina *in-vivo*. **f**, Spectral binning to divide the swept wavelength into 9 windows. **g**. The angles between the average Stokes vectors measured from the orange area indicated in **e**. The values are not exactly 90° due to the existence of diattenuation of the system and

sample. **h**, Theoretical bonds of the angle between two orthogonal Stokes vectors after travelling through a diattenuating medium. **i**, Orthogonality of the Stokes vectors of the backscattered light from the retinal images in **e** indicated by the orange area before and after corneal diattenuation correction.

We used the image of a guinea pig retina *in-vivo* to test the modulation performance. We calculated the averaged Stokes vector in a region in the inner retina, indicated in the orange area in **Supplementary Method Fig. 2e**. In the wavelength domain, the fringe was divided into 9 partially overlapped bins, as shown in **Supplementary Method Fig. 2f**. For each bin, we calculated the angles between the 3 Stokes vectors. Shown in **Supplementary Method Fig. 2g**, we find that the maximum error is approximately ±10° to the set value of 90°. Judging from the theoretical bonds of the angle between two orthogonal Stokes vectors after travelling through a diattenuating medium (**Supplementary Method Fig. 2h**), the magnitude of system and sample attenuation, ***D***, should be around 0.1, defined as the relative difference between the maximum (p_1^2) and minimum (p_2^2) attenuation coefficients, where $D = (p_1^2 - p_2^2)/(p_1^2 + p_2^2)$. The variation between bins is due to the polarization mode dispersion and the wavelength dependence of the polarizing beam splitter. After applying the proposed method to correct for diattenuation using the signals of the surface of the retina, the orthogonality of the Stokes vectors is restored with an error of less than 3° (**Supplementary Method Fig. 2i**).

Supplementary Method 4: Recovery of the reciprocal constraint for depth cumulative Mueller matrices

In a backscattering OCT detection system, there is a reciprocal light path, or double traveling path, where the probing light travels into the sample and then travels back to the detection system through the same path after being backscattered by the sample internal structures. The reciprocal light path adds a symmetry constraint to the detected Mueller matrix[15]. Taking advantage of the symmetry constraint, the free parameters in the Mueller matrix are further reduced, and the depth-resolved physical optic axis can be solved with a prior knowledge of the underlying structure i.e., Henle fiber layer. The reciprocity of light in our TRIPS-OCT system is disrupted by the non-reciprocally traversed optical circuit induced by a circulator[16]. The circulator guides the probing light from the source to the sample and guides the backscattered light to the detection unit. However, the reciprocal symmetry can be recovered by a compensation Mueller matrix, $\mathbf{A}$, describing the polarization properties of an asymmetric medium in the system. The method to solve $\mathbf{A}$ was developed by Villiger *et al.*[15] and Li *et al.*[17].

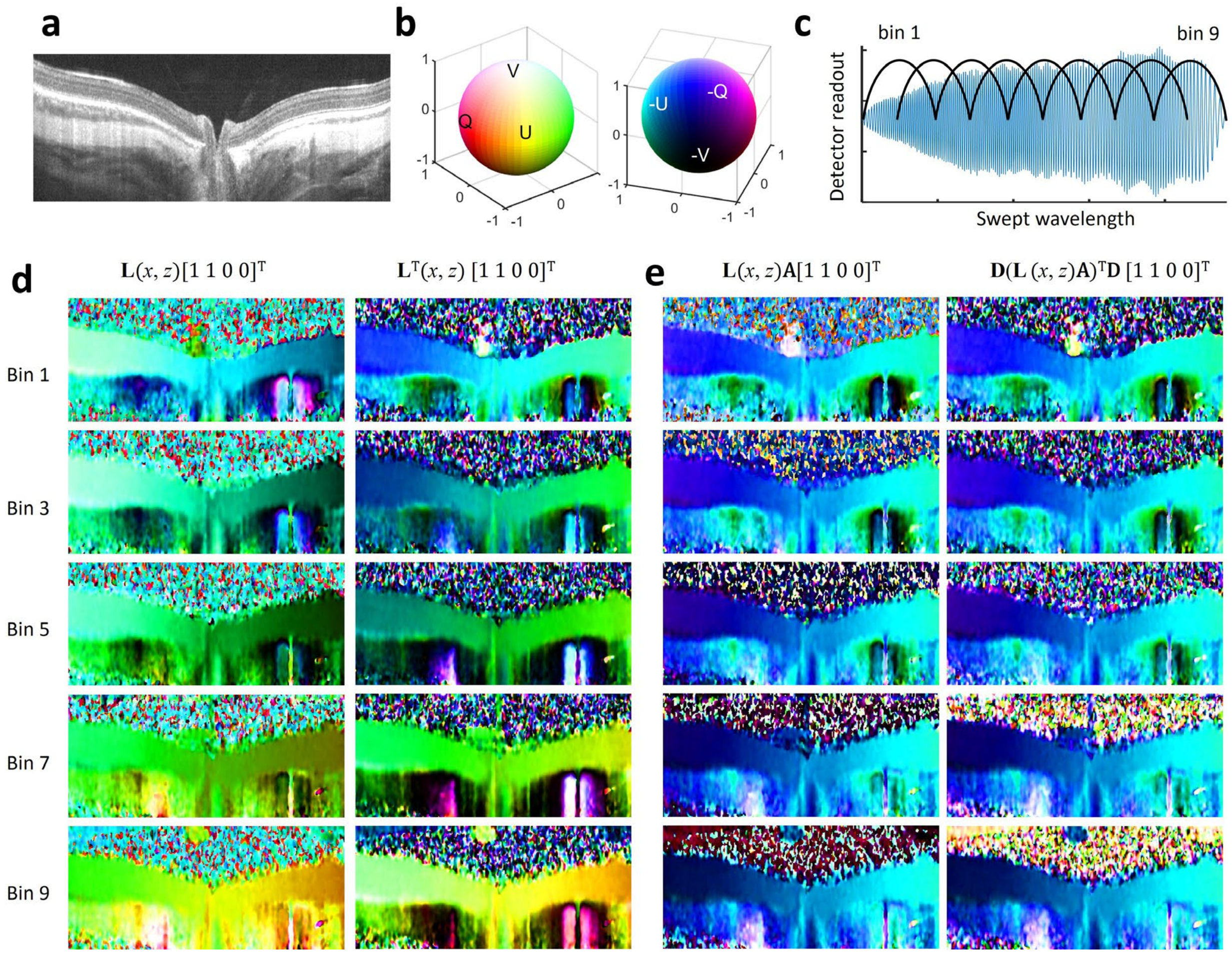

Supplementary Method Fig. 3 | Recovery of the reciprocal constraint for depth cumulative Mueller matrices. **a**, B-scan image of a guinea pig retina *in-vivo*. **b**, Color-coding of the Poincaré sphere. **c**, Spectral binning. **d**, Normalized Stokes vectors in the image before compensation. **e**, Normalized Stokes vectors in the image after compensation.

Briefly, the depth cumulative Mueller matrix of a sample, $\mathbf{M}$, measured from a reciprocal system, has a symmetry constraint, expressed as $\mathbf{M} = \mathbf{D}\mathbf{M}^\mathrm{T}\mathbf{D}$, where $^\mathrm{T}$ represents the transpose operation, $\mathbf{D} = \mathrm{diag}(1,1,1,-1)$. Due to the circulator, the original measured Mueller matrix, $\mathbf{L}$, does not fulfil the symmetry constraint. The compensation Mueller matrix, $\mathbf{A}$, can recover the symmetry constraint, expressed as $\mathbf{LA} = \mathbf{D}(\mathbf{LA})^\mathrm{T}\mathbf{D}$. In the TRIPS-OCT measurement, the Mueller matrix $\mathbf{L}$ for every pixel in each spectral bin is reconstructed, denoted as $\mathbf{L}(x, y, z)$, where x, y are the lateral directions and z is the depth. We randomly pick 10 frames from the volume and select pixels with an intensity signal larger than 10 dB. The Muller matrices of those pixels are denoted as $\mathbf{L}(s)$, where s is the index of the selected pixels. $\mathbf{A}$ is solved with an optimization problem, formulated as

$$\min \sum_s \|\mathbf{LA} - \mathbf{D}(\mathbf{LA})^{\mathrm{T}}\mathbf{D}\|.$$

Note that $\mathbf{A}$ is a pure Mueller matrix. The minimization problem can be further formulated as a matrix eigenvalue problem for analytical solution[15] or solved numerically.

In the TRIPS-OCT system, $\mathbf{A}$ is a fixed matrix for one volume scan for each spectral bin. To test the performance of the recovery of the reciprocal constraint. We used one B-scan image of a guinea pig retina *in-vivo* (**Supplementary Method Fig. 3a**). We color-coded the Poincaré sphere, specifically, representing one polarization state with one unique color on the Poincaré sphere (**Supplementary Method Fig. 3b**). Spectral binning is performed (**Supplementary Method Fig. 3c**), and the compensation matrix $\mathbf{A}$ is solved for each bin. As shown in **Supplementary Method Fig. 3d**, we use a sampling matrix $[1,1,0,0]^{\mathrm{T}}$ to generate the Stokes vectors from the reconstructed Mueller matrices in this frame, denoted as $\mathbf{L}(x,z)$. Before the compensation, the Stokes vectors are obviously different, generated from the original Mueller matrix $\mathbf{L}(x,z)$ and the reciprocal symmetric counterpart $\mathbf{DL}(x,z)^{\mathrm{T}}\mathbf{D}$. After compensating for $\mathbf{A}$, the reciprocal symmetry is recovered with similar Stokes vectors, as shown in **Supplementary Method Fig 3e**.

The reciprocal constraint was forced to the compensated volume Mueller matrices, $\mathbf{L}_c(x,y,z,b)$, by

$$\mathbf{L}_c(x,y,z,b) = \frac{\mathbf{L}(x,y,z,b)\mathbf{A}(b) + \mathbf{DA}(b)^{\mathrm{T}}\mathbf{L}(x,y,z,b)^{\mathrm{T}}\mathbf{D}}{2},$$

where b is the index of the spectral bin, $b \in \{1,2 \ldots ,9\}$ in our system.

Supplementary Method 5: Spectral binning to remove wavelength-dependent polarization variation

Due to the wavelength-dependent variation of the laser source and optical components, it is inevitable to induce polarization mode dispersion (PMD) into a PS-OCT system[18,19]. Spectral binning to mitigate PMD was proposed[20] by dividing the fringe into several windows, in which the PMD is small and negligible. The variation of the polarization proprieties of a spectral bin to the central bin can be described by similarity transformation with a pure 4×4 Mueller matrix. In this manner, a minimization problem to define the similarity transformation can be formulated as

$$\min \sum_{b-scans} \|\mathbf{B}(b)\mathbf{L}(x,y,z,b)\mathbf{B}(b)^{-1} - \mathbf{L}(c)\|.$$

where b is the bin index, c is the index of the central bin, x, y are the lateral directions and z is the depth. The minimization problem can be solved numerically within randomly picked frames in a volume scan.

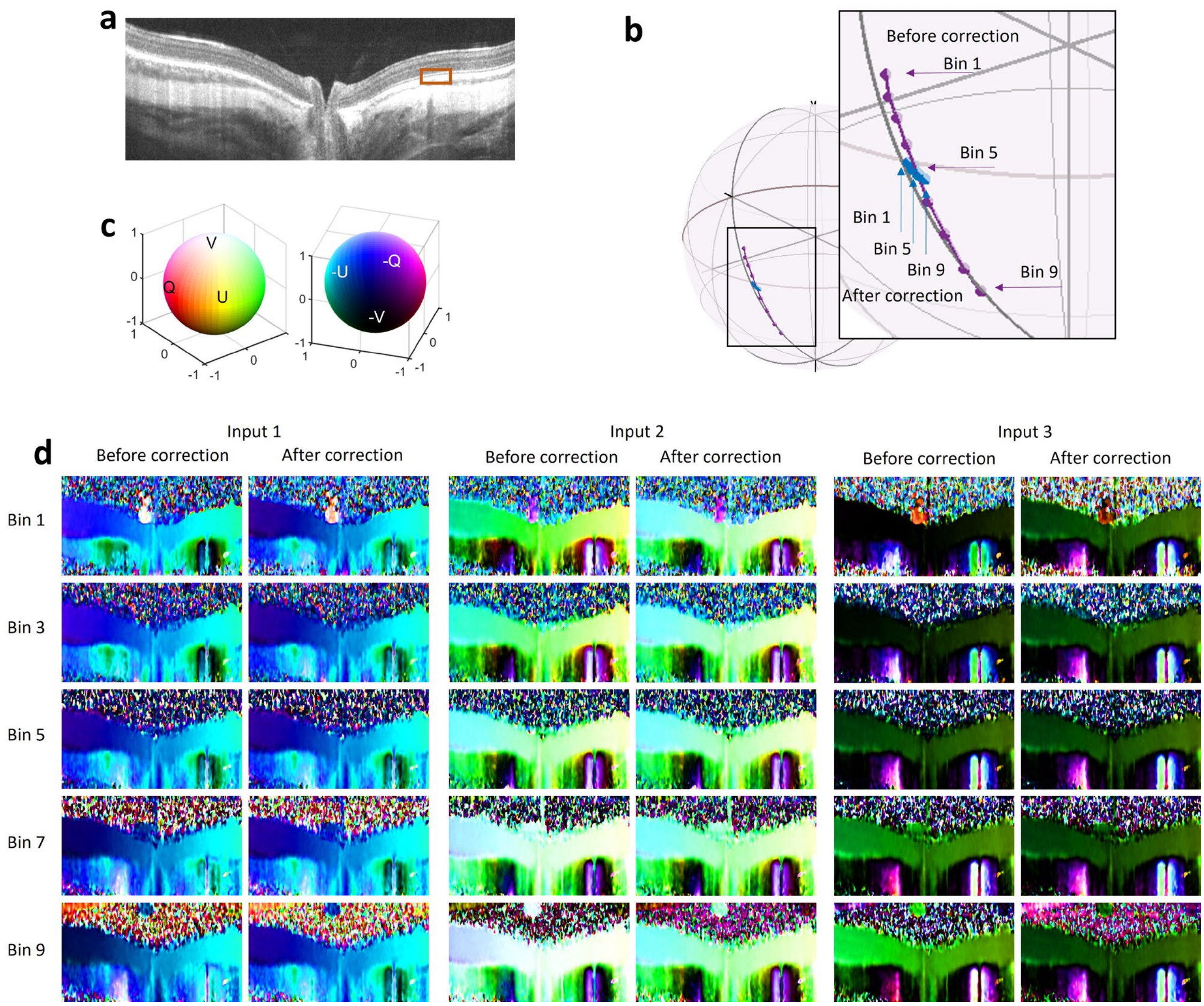

Supplementary Method Fig. 4 | Spectral binning to remove wavelength-dependent polarization variation (polarization mode dispersion). a, B-scan image of a guinea pig retina *in-vivo*. **b**, Normalized averaged Stokes vector before and after PMD correction in the area indicated in **a** from different spectral bins. **c**, Color-coding of the Poincaré sphere. **c**, Spectral binning. **d**, Normalized Stokes vectors in the image before and after compensation.

To characterize the PMD in the TRIPS-OCT system, we randomly selected an area in a B-scan of a guinea pig retina, indicated in the orange box in **Supplementary Method Fig. 4a**. We averaged the Stokes vectors in this region from different bins. The normalized Stokes vectors from 9 bins are plotted on the Poincaré sphere

in **Supplementary Method Fig. 4b** (purple dot and purple line). The spread of the polarization states between different bins suggests the existence of PMD in the system. To test the PMD removal correction, we applied the above correction to the Mueller matrices and plotted the corrected Stokes vectors (blue dot and purple line). It can be observed that PMD was significantly reduced by observing the spread of the polarization states on the Poincaré sphere. To test the performance on a B-scan image, we used color-coded polarization states (**Supplementary Method Fig. 4c**) to represent the normalized Stokes vectors in a B-scan. As a triple input system, we checked the triple-input polarization states by multiplying the Mueller matrices with $[1,1,0,0]^{\mathrm{T}}$, $[1,0,1,0]^{\mathrm{T}}$, and $[1,0,0,1]^{\mathrm{T}}$. Comparing the images from different bins (**Supplementary Method Fig. 4d**), we find that the PMD is substantially removed using the proposed method.

Finally, the Mueller matrices from the 9 bins were elementwise averaged to obtain a general Mueller matrix volume, expressed as

$$\mathbf{M}(x,y,z) = \frac{\sum_b \mathbf{L}_c(x,y,z,b)}{n},$$

where $\mathbf{M}(x,y,z)$ is a general Mueller matrix volume and b is the index of spectral bins. n is the total number of bins, and x, y, and z are the coordinates along the fast and slow scan directions and depth. $\mathbf{L}_c(x,y,z,b)$ is the volume Mueller matrices obtained from **Supplementary Method 4**.

To remove the depolarization component in $\mathbf{M}(x,y,z)$, polar decomposition[21] was used, as

$$\mathbf{M}(x,y,z) = \mathbf{M}_\Delta(x,y,z)\mathbf{M}_R(x,y,z)\mathbf{M}_D(x,y,z)$$

where $\mathbf{M}_\Delta(x,y,z)$ is a depolarizer, $\mathbf{M}_R(x,y,z)$ is a retarder and $\mathbf{M}_D(x,y,z)$ is a diattenuator. Combined, $\mathbf{M}_P(x,y,z) = \mathbf{M}_R(x,y,z)\mathbf{M}_D(x,y,z)$ defines the pure Mueller matrix of the cumulative round-trip to sample depth z. Using the spectral binning correction, the polarization mode dispersion of the optical components and samples, as well as wavelength dependent splitting of the PBSs in the detection unit can be mitigated.

Supplementary Method 6: Compensation of corneal retardance and diattenuation

The cornea is birefringent[22]. The cornea-air interface may induce attenuation when the beam is entering and leaving the eye.

We compensated for the birefringence of the cornea in a frame-based manner. The surface of the retina was detected (indicated by the red line in **Supplementary Method Fig. 5a**). The Mueller matrices on the surface were extracted, denoted as $\mathbf{S}(x)$, which is a function of the lateral position. To reconstruct the single pass cornea birefringence, $\mathbf{C}(x)$, the differential Mueller matrix $\mathbf{s}(x)$ was calculated by taking the matrix logarithm of $\mathbf{S}(x)$. The retardance vector $\mathbf{r}(x) = [r_1\ r_2\ r_3]^{\mathrm{T}}$ and diattenuation vector $\mathbf{d}(x) = [d_1\ d_2\ d_3]^{\mathrm{T}}$ can be obtained from entries of $\mathbf{s}(x)$, expressed as

$$\mathbf{s} = \begin{bmatrix} 0 & d_1 & d_2 & d_3 \\ d_1 & 0 & r_3 & -r_2 \\ d_2 & -r_3 & 0 & r_1 \\ d_3 & r_2 & -r_1 & 0 \end{bmatrix}.$$

Considering the wrapping effect, the birefringence of the cornea, $\mathbf{b}(x)$, specifically, the linear retardance and linear diattenuation, can be represented with the complex vector, noted as

$$\mathbf{b}(x) = \mathbf{r}(x) - i\mathbf{d}(x) + 2n\pi\left(\frac{\mathbf{r}(x) - i\mathbf{d}(x)}{|\mathbf{r}(x) - i\mathbf{d}(x)|}\right),$$

where n is an integer. By choosing n, we can unwrap $\mathbf{b}(x)$ to force continuity of the corneal retarder and diattenuator, not only in the *x* direction but also in the *x-y* plane considering the slow scan direction, as shown in **Supplementary Method Figs. 5b,c**.

After unwrapping $\mathbf{r}(x)$ and $\mathbf{d}(x)$, the single pass cornea birefringence $\mathbf{C}(x)$ can be reconstructed by a Mueller matrix representing the retarder $\mathbf{r}(x)/2$ and $\mathbf{d}(x)/2$. The linear retardance and diattenuation effect of the system and the cornea was then compensated by$\mathbf{M}_{PC}(x,z) = \mathbf{C}^{-1}(x)\mathbf{M}_P(x,z)\mathbf{C}^{-1}(x)$, where $\mathbf{M}_{PC}(x,z)$ is the compensated cumulative round-trip Mueller matrix.

To test the performance of cornea compensation, we show the polarization states of a retina cross-sectional image with color coded the Poincaré sphere. As shown in **Supplementary Method Fig. 5d**, the polarization state evolution along the lateral direction in the inner retina is due to corneal birefringence. After compensation, we observe that the polarization state along the lateral direction is constant, indicating that the effect of corneal birefringence is removed (**Supplementary Method Fig. 5e**). To test the effect of unwrapping, we construct optic axis images of the sclera with and without unwrapping. If not unwrapped, we observe that an artifact is created in the optic axis image (**Supplementary Method Fig. 5f**).

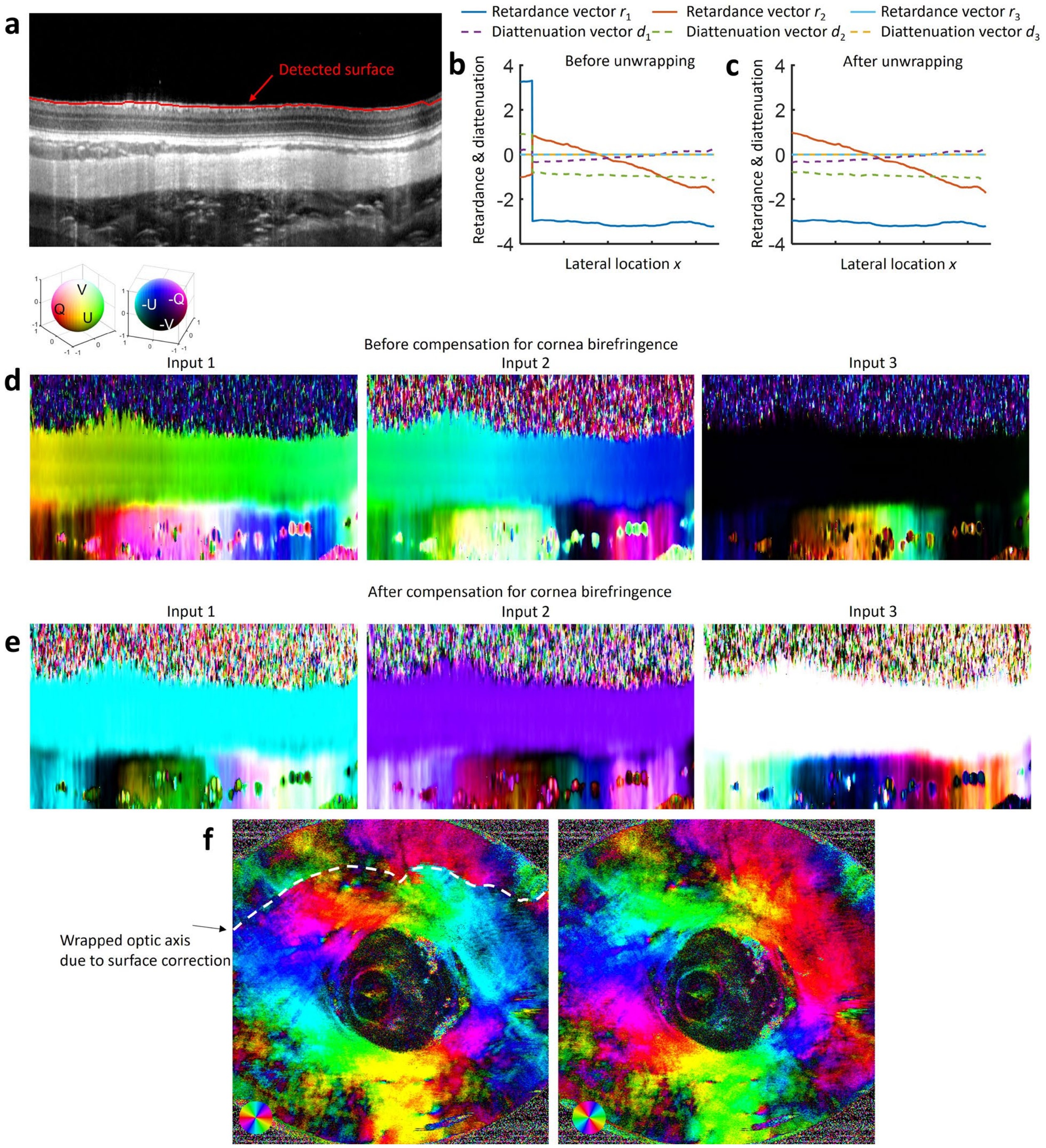

Supplementary Method Fig. 5 | Compensation of corneal retardance and diattenuation. a, B-scan image of a guinea pig retina *in-vivo*. The red line indicates the surface of the retina. **b**, **c**, Retardance and diattenuation vectors of corneal birefringence before (**b**) and after unwrapping (**c**). **d**, **e**, Polarization states of retinal cross-sectional image using the Poincaré sphere color-coding before (**d**) and after (**e**) cornea compensation. **f**, Optic axis images of the sclera with and without unwrapping.

Supplementary Method 7: Validation of depth-resolved optic axis measurement using a phantom

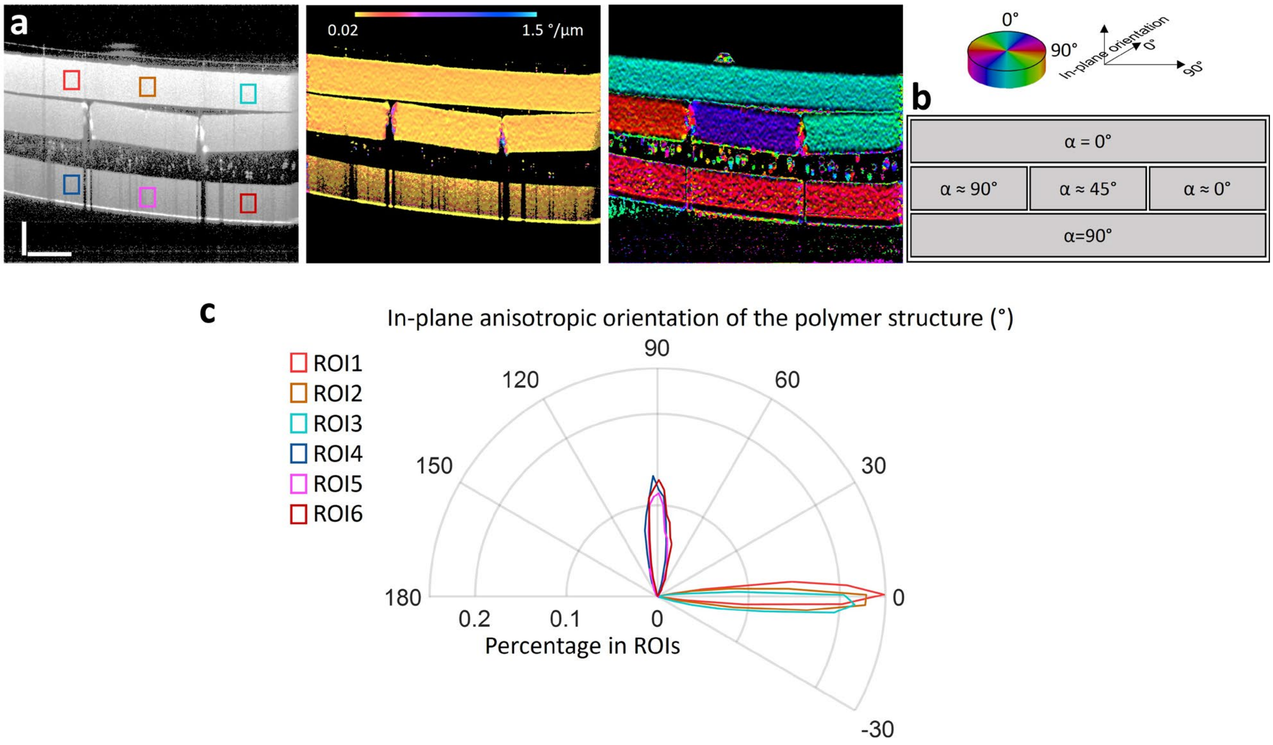

Supplementary Method Fig. 6 | Validation of depth-resolved optic axis measurement using a phantom. **a**, TRIPS-OCT depth-resolved optic axis imaging on a phantom. **b**, True phantom architecture. **c**, Angular histograms of measured optic axis orientation in multiple regiosn of interest (ROIs) from the cross-sectional images indicated in **a**. Scale bars, **a**, vetical: 250 µm, horizontal: 1 mm.

We used a phantom to validate the depth-reolved optic axis reconstruction using TRIPS-OCT (**Supplementary Method Fig. 6a**). The phantom contains three layers of 250-µm acrylonitrile butadiene styrene (ABS) sheet (McMASTER-CARR, CA, US). Homogeneous birefringence with a uniform optic axis was present in the material due to the extrusion manufacture process. The anisotropic orientation of the polymer structure, noted as α, can be measured under TRIPS-OCT as the optic axis orientation. A phatom[23] with a designed architecture (**Supplementary Method Fig. 6b**) can be assembled by cutting the ABS sheet into small pieces. The absolute 0° was set as the optic axis orientation measured in ROI1. From the angular histograms (**Supplementary Method Fig. 6c**), in the first layer, the stardand deviation of orientation measurement is 4.6°; in the third layer, the stardand deviation of orientation measurement is 11.5°. Overall, from the measurement of the thrid layer of the phatom, TRIPS-OCT demonstrates a good performace of depth-resolved optic axis measurement independent of superficial sample structures.

Supplementary Method 8: Enface birefringence image projection in guinea pigs and humans

The strategies for the guinea pig model and humans to obtain *enface* birefringence images of the sclera are different due to the difference of eye sizes.

For guinea pigs, we used a maximum projection in the *enface* direction to minimize the influence of varying tilts in different scans. Before performing the maximum projection, the depolarization index, P_Δ, is calculated from the pixel-based Mueller matrix, expressed as

$$P_\Delta = \sqrt{\frac{\mathrm{tr}\left(\widehat{\mathbf{M}}^{\mathrm{T}}\widehat{\mathbf{M}}\right) - 1}{3}},$$

where $\widehat{\mathbf{M}} = \mathbf{M}/m_{00}$, m_{00} is the first entry of the Mueller matrix, and $\mathrm{tr}(\cdot)$ represents the trace of a matrix. As shown in **Supplementary Method Fig. 7a**, we used 0.9 as a threshold to create a binary map of the depolarization index to remove the noise and structures inducing depolarization in the retina. We used a 30 µm vertical line kernel to filter each cross-sectional birefringence image and then performed the maximum projection along the depth.

For humans, we manually label the choroidal-scleral interface on the intensity image. As shown in **Supplementary Method Fig. 7b**, to obtain the *en-face* birefringence image, we use a 200 µm slab on the cross-sectional image centered on the choroidal-scleral interface 100 µm above and 100 µm below to include fine structures on the scleral surface and calculate the mean values along the lateral direction. The scleral birefringence deeper than 100 µm from the choroidal-scleral interface may not be reliable because of the strong signal attenuation in the choroid and sclera structures.

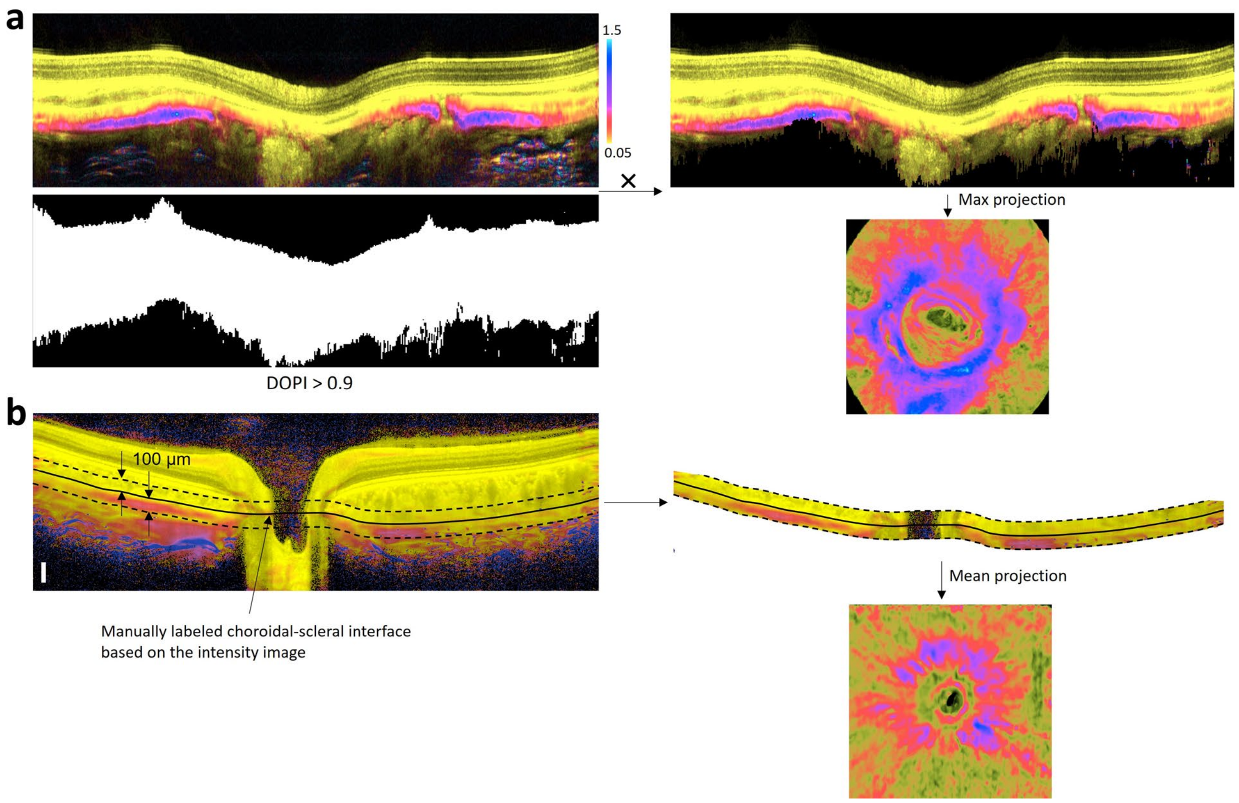

Supplementary Method Fig. 7 | Image processing to obtain enface birefringence images in guinea pigs (a) and humans (b).

Supplementary Method 9: Human retina imaging interface

We display an image on a 7-inch LCD screen to help the users fix their eyes. The image of the screen is coupled to the scanning optics using a longpass dichronic mirror (DMLP650L, Thorlabs, Newton, US) (**Supplementary Method Fig. 8a**). The scanning optics are compactly built on the top of a joystick-controlled motion platform (**Supplementary Method Fig. 8b**). When performing the scanning, the users place their head on the chin rest and look at the fixation target, while the operator positions the platform according to the preview of the retina B-scan (**Supplementary Method Fig. 8c**). Two alignment cameras are used to help in the positioning. The segmentation of the retina image is performed with a custom developed app, as shown in **Supplementary Method Fig. 8d**, where the user can place and adjust the positions of the key points to define a spline in an interactive interface.

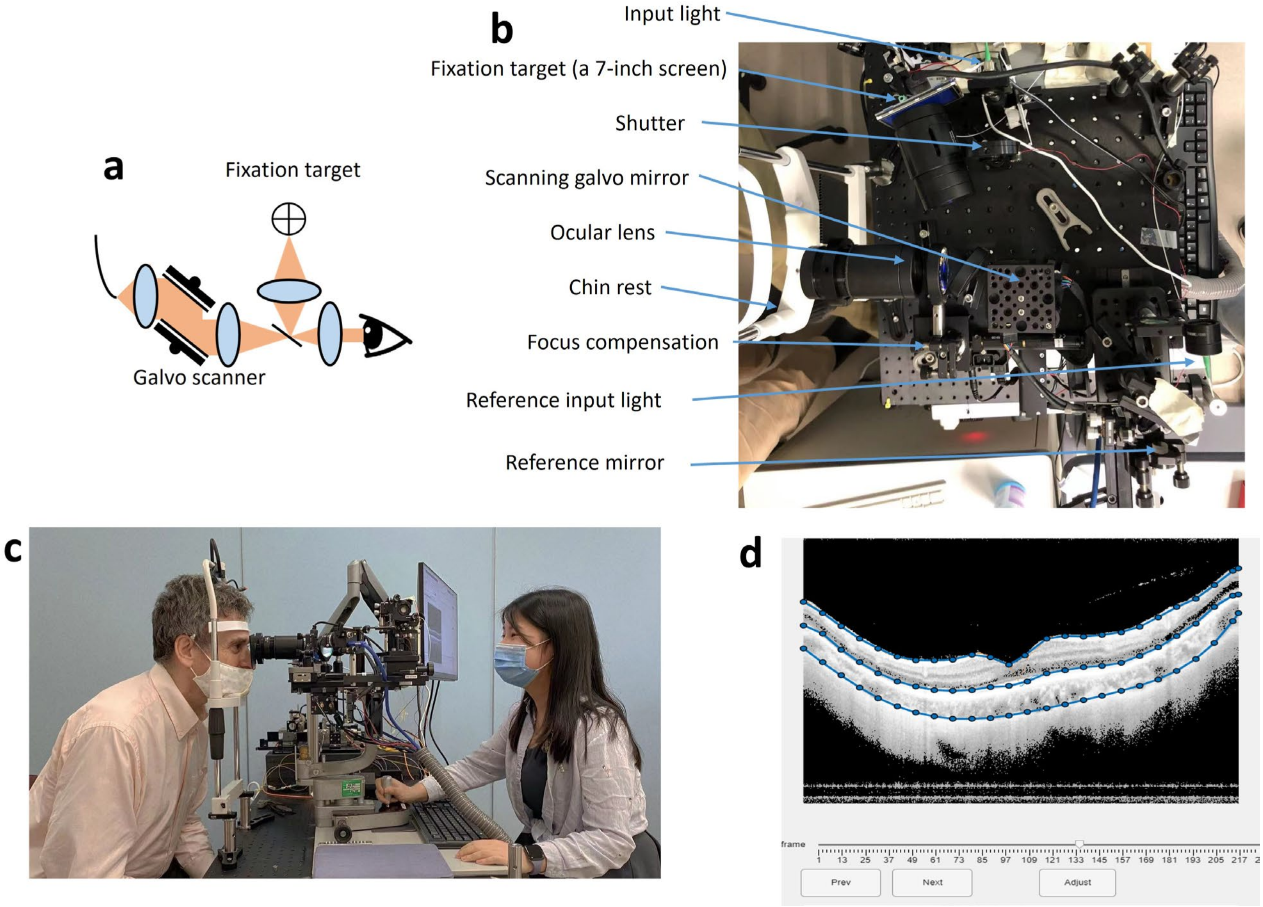

Supplementary Method Fig. 8 | Human retina imaging interface. a, Schematic of the fixation target. **b**, Compact scanning optics on a motion platform. **c**, Two scientists demonstrate the usage of the TRIPS-OCT system to operators. **d**, Retina segmentation software.

Supplementary Data 1: In-vivo TRIPS-OCT images of a guinea pig at 16-week-old before TEM analysis

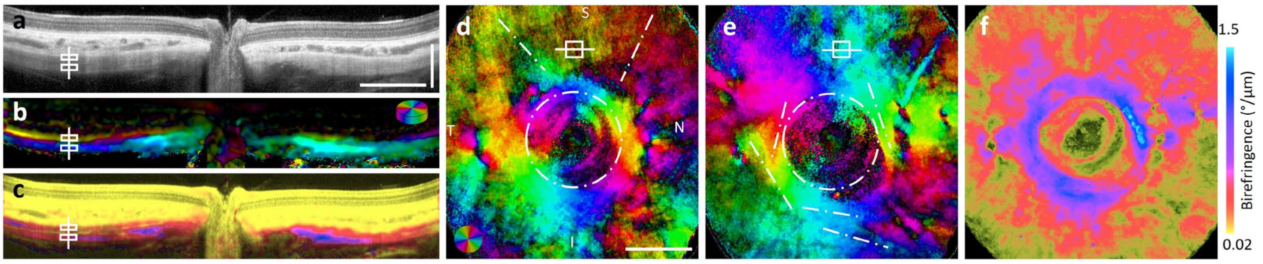

Supplementary Data Fig. 1 | TRIPS-OCT images of a guinea pig at 16-week-old. a-c, Cross-sectional images of a guinea pig eye under intensity (**a**), optic axis (**b**) and birefringence contrast (**c**). **d**, **e**, *En face* optic axis images of the inner (**d**) and outer (**e**) sclera. White dotted lines indicate local fiber orientations. White boxes indicate the approximate locations (inner and outer sclera) of TEM analysis. The white line indicates the sectioning direction. **f**, *En-face* maximum birefringence projection of the entire sclera.

Supplementary Data 2: Longitudinal observations of scleral birefringence development during refraction development in guinea pigs

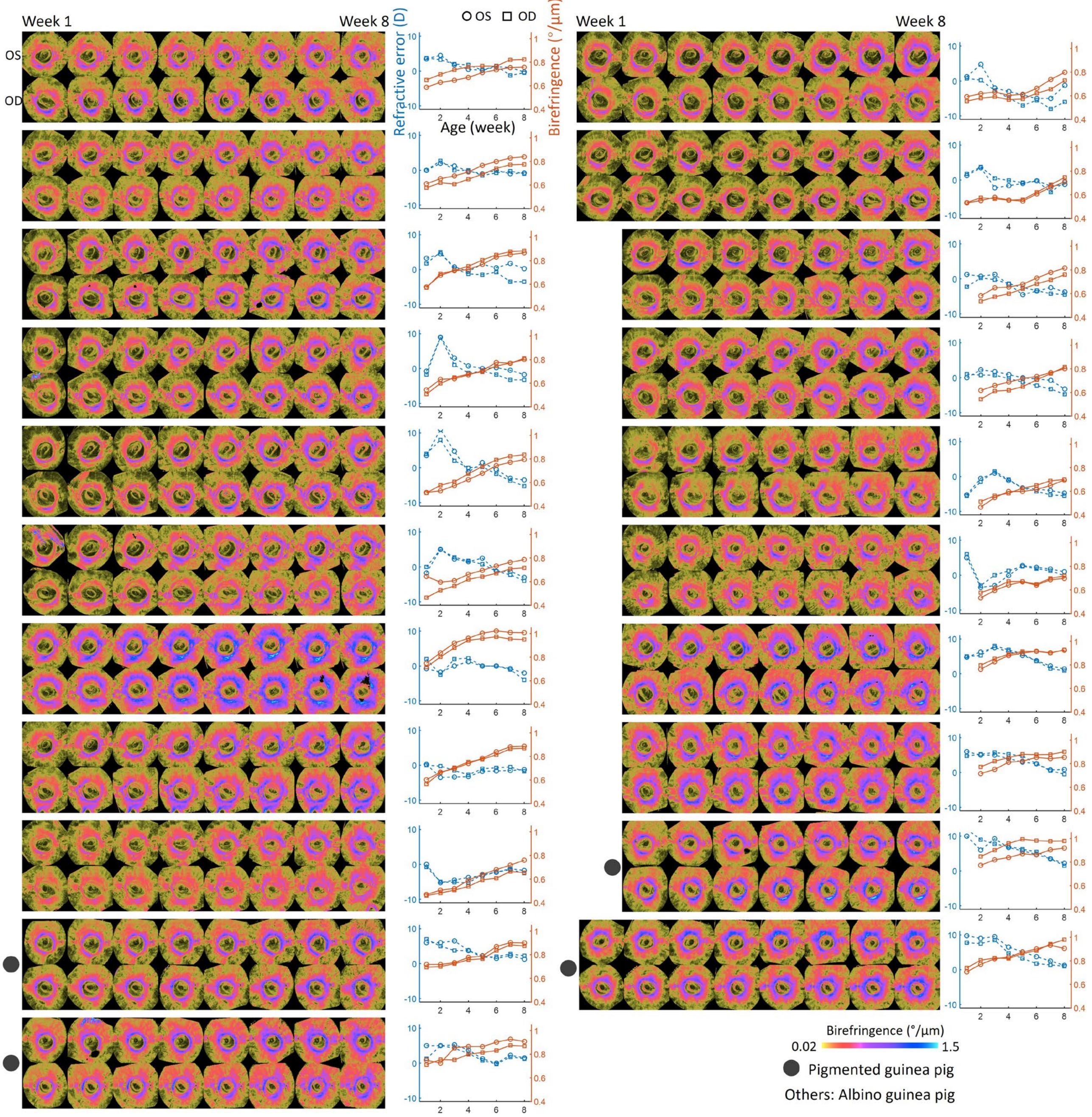

Supplementary Data Fig. 2 | TRIPS-OCT *en-face* scleral birefringence images, measured scleral birefringence values, and refractive errors in 21 guinea pigs.

Supplementary Data 3: Scleral birefringence and refractive error in guinea pigs from ages of 2-8 weeks

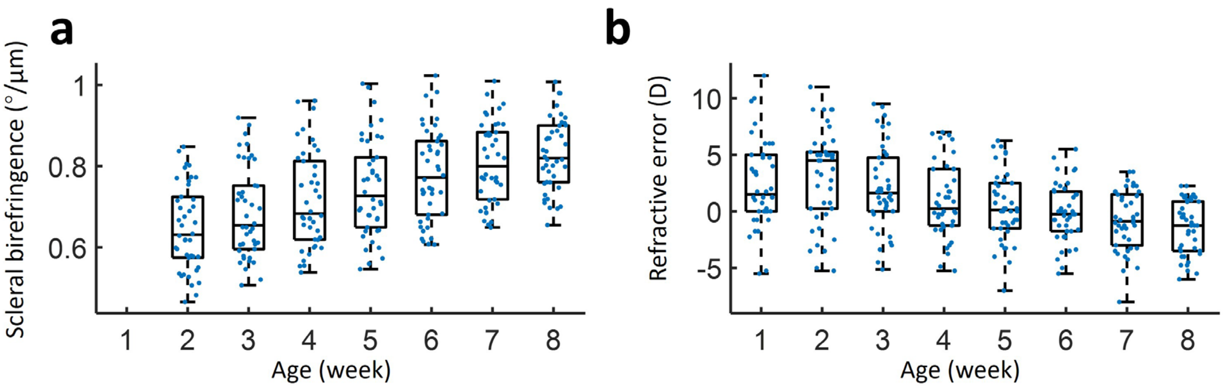

Supplementary Data Fig. 3 | Measured scleral birefringence values and refractive errors at ages from 2-8 weeks. Dots represent n = 42 eyes from 21 guinea pigs, central line indicates median, box shows interquartile range and whiskers show range.

Supplementary Data 4: Location dependence of scleral birefringence and myopia status in the emmetropia and low myopia group

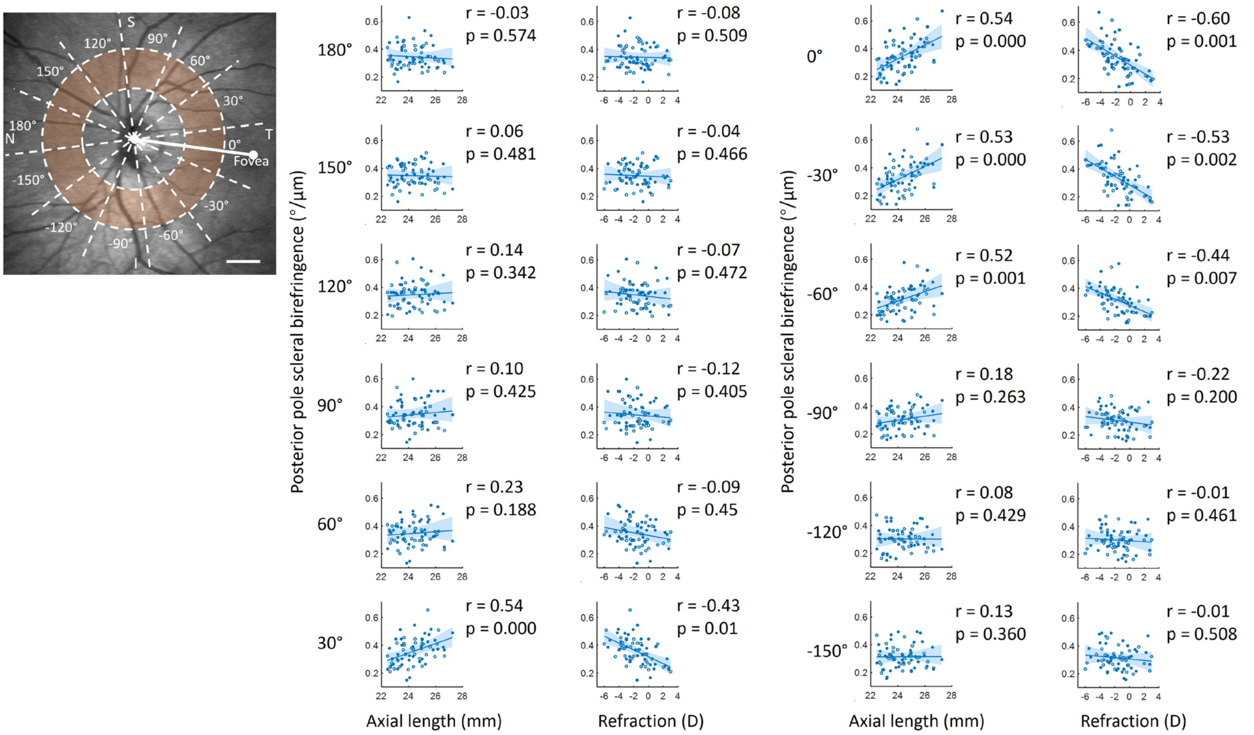

Supplementary Data Fig. 4 | Correlation analysis of different retinal locations between posterior scleral birefringence and refraction, axial length in eyes in the emmetropia and low myopia group. Scatter plots show 69 eyes from 42 individuals, regression (lines) and 95% confidence intervals (shaded areas). The *r* values are calculated by Pearson correlation. The *p* values are calculated by F-test against a constant model. Inter-eye correlation is addressed by bootstrapping.

Supplementary Data 5: Correlation between biometrics of the human eyes in the emmetropia and low myopia group

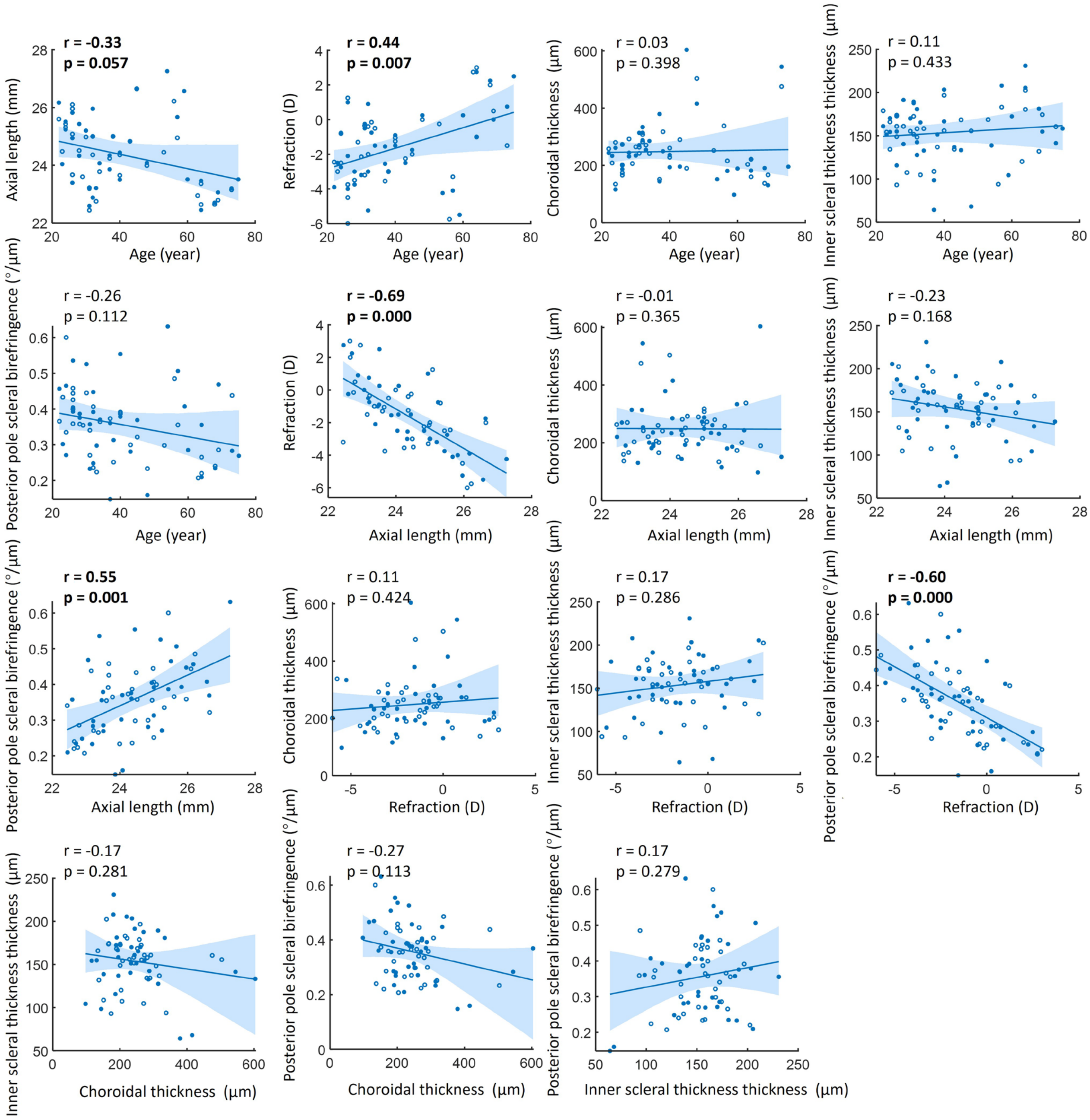

Supplementary Data Fig. 5 | Correlation analysis of age, axial length, choroidal thickness, inner scleral thickness and posterior pole scleral birefringence in the eyes in the emmetropia and low myopia group. Scatter plots show 69 eyes from 42 individuals, regression (lines) and 95% confidence intervals (shaded areas). r values are calculated by Pearson correlation. p values are calculated by F-test against a constant model. Inter-eye correlation is addressed by bootstrapping.

Supplementary Data 6: Grouping of eyes and subjects and characteristics

Supplementary Table. 1 | The characteristics of eyes and subjects in three groups. SD: Standard deviation.

Group 1: Emmetropia or low myopia (>-6D, ≤3D), no pathological conditions		
Factor	Mean	SD
Age (year)	41.29	16.03
Sex	Female N=23, Male N=19	
Refractive error (D)	-1.74	2.31
Axial length (mm)	24.44	1.26
Group 2: High myopia (<= -6D), no pathological conditions		
Age (year)	39.20	10.51
Sex	Female N=8, Male N=1	
Refractive error (D)	-7.72	1.10
Axial length (mm)	26.88	1.18
Group 3: Pathologic myopia with staphyloma		
Age (year)	58.22	6.69
Sex	Female N=6, Male N=3	
Axial length (mm)	29.05	2.10

References

1. Haskell, R. C., Carlson, F. D. & Blank, P. S. Form birefringence of muscle. *Biophys. J.* **56**, 401–413 (1989).
2. Tseng, S. H. *et al.* Corneal transparency and scleral opacity arises from the nanoarchitecture of the constituent collagen fibrils. *Biomed. Opt. Express* **13**, 1485 (2022).
3. Keeley, F. W., Morin, J. D. & Vesely, S. Characterization of collagen from normal human sclera. *Exp. Eye Res.* **39**, 533–542 (1984).
4. Komai, Y. & Ushiki, T. The three-dimensional organization of collagen fibrils in the human cornea and sclera. *Investig. Ophthalmol. Vis. Sci.* **32**, 2244–2258 (1991).
5. Meek, K. . & Fullwood, N. . Corneal and scleral collagens—a microscopist's perspective. *Micron* **32**, 261–272 (2001).
6. Spiesz, E. M., Thorpe, C. T., Thurner, P. J. & Screen, H. R. C. Structure and collagen crimp patterns of functionally distinct equine tendons, revealed by quantitative polarised light microscopy (qPLM). *Acta Biomater.* **70**, 281–292 (2018).
7. McBrien, N. A., Cornell, L. M. & Gentle, A. Structural and ultrastructural changes to the sclera in a mammalian model of high myopia. *Investig. Ophthalmol. Vis. Sci.* **42**, 2179–2187 (2001).
8. American National Standards Institute, I. *American National Standard for Ophthalmics – Light Hazard Protection for Ophthalmic Instruments*. *The Vision Council* (2021).
9. Yamanari, M. *et al.* Melanin concentration and depolarization metrics measurement by polarization-sensitive optical coherence tomography. *Sci. Rep.* **10**, (2020).
10. Lippok, N. *et al.* Depolarization signatures map gold nanorods within biological tissue. *Nat. Photonics* **11**, 583–588 (2017).
11. Anderson, D. G. M. & Barakat, R. Necessary and sufficient conditions for a Mueller matrix to be derivable from a Jones matrix. *J. Opt. Soc. Am. A* **11**, 2305 (1994).
12. Villiger, M. *et al.* Spectral binning for mitigation of polarization mode dispersion artifacts in catheter-based optical frequency domain imaging. *Opt. Express* **21**, 16353–16369 (2013).
13. Xiong, Q. *et al.* Constrained polarization evolution simplifies depth-resolved retardation measurements with polarization-sensitive optical coherence tomography. *Biomed. Opt. Express* **10**, 5207 (2019).
14. Willemse, J. *et al.* Optic axis uniformity as a metric to improve the contrast of birefringent structures and analyze the retinal nerve fiber layer in polarization-sensitive optical coherence tomography. *Opt. Lett.* **44**, 3893 (2019).
15. Villiger, M. *et al.* Optic axis mapping with catheter-based polarization-sensitive optical coherence tomography. *Optica* **5**, 1329 (2018).
16. Park, B. H., Pierce, M. C., Cense, B. & de Boer, J. F. Optic axis determination accuracy for fiber-based polarization-sensitive optical coherence tomography. *Opt. Lett.* **30**, 2587 (2005).
17. Li, Q. *et al.* Robust reconstruction of local optic axis orientation with fiber-based polarization-sensitive optical coherence tomography. *Biomed. Opt. Express* **9**, 5437 (2018).
18. Gordon, J. P. & Kogelnik, H. PMD fundamentals: polarization mode dispersion in optical fibers. *Proc. Natl. Acad. Sci. U. S. A.* **97**, 4541–4550 (2000).
19. Villiger, M. *et al.* Artifacts in polarization-sensitive optical coherence tomography caused by polarization mode dispersion. *Opt. Lett.* **38**, 923–925 (2013).
20. Villiger, M. *et al.* Spectral binning for mitigation of polarization mode dispersion artifacts in catheter-based optical frequency domain imaging. *Opt. Express* **21**, 16353–16369 (2013).
21. Lu, S.-Y. & Chipman, R. A. Interpretation of Mueller matrices based on polar decomposition. *J. Opt. Soc. Am. A* **13**, 1106 (1996).
22. Pircher, M. *et al.* Corneal birefringence compensation for polarization sensitive optical coherence tomography of the human retina. *J. Biomed. Opt.* **12**, 041210 (2007).
23. Liu, X. *et al.* Tissue-like phantoms for quantitative birefringence imaging. *Biomed Opt Express* **8**, 4454 (2017).
