# Supplementary material for: Posterior scleral birefringence measured by triple-input polarization-sensitive imaging as a biomarker of myopia progression
Source: Nat Biomed Eng. 2023 Jun 26;7(8):986–1000. doi: 10.1038/s41551-023-01062-w (PMC10427432; doi:10.1038/s41551-023-01062-w)
Supplement: Supplementary file 2 — Reporting Summary [file 41551_2023_1062_MOESM2_ESM.pdf]

nature portfolio

Corresponding author(s): Leopold Schmetterer

Last updated by author(s): Mar 23, 2023

Reporting Summary

Nature Portfolio wishes to improve the reproducibility of the work that we publish. This form provides structure for consistency and transparency in reporting. For further information on Nature Portfolio policies, see our Editorial Policies and the Editorial Policy Checklist.

Statistics

For all statistical analyses, confirm that the following items are present in the figure legend, table legend, main text, or Methods section.

n/a	Confirmed	
☐	☒	The exact sample size (n) for each experimental group/condition, given as a discrete number and unit of measurement
☐	☒	A statement on whether measurements were taken from distinct samples or whether the same sample was measured repeatedly
☐	☒	The statistical test(s) used AND whether they are one- or two-sided<br>*Only common tests should be described solely by name; describe more complex techniques in the Methods section.*
☒	☐	A description of all covariates tested
☐	☒	A description of any assumptions or corrections, such as tests of normality and adjustment for multiple comparisons
☐	☒	A full description of the statistical parameters including central tendency (e.g. means) or other basic estimates (e.g. regression coefficient) AND variation (e.g. standard deviation) or associated estimates of uncertainty (e.g. confidence intervals)
☐	☒	For null hypothesis testing, the test statistic (e.g. F, t, r) with confidence intervals, effect sizes, degrees of freedom and P value noted<br>*Give P values as exact values whenever suitable.*
☒	☐	For Bayesian analysis, information on the choice of priors and Markov chain Monte Carlo settings
☒	☐	For hierarchical and complex designs, identification of the appropriate level for tests and full reporting of outcomes
☐	☒	Estimates of effect sizes (e.g. Cohen's d, Pearson's r), indicating how they were calculated

Our web collection on statistics for biologists contains articles on many of the points above.

Software and code

Policy information about availability of computer code

Data collection	NI LabVIEW 2020 was used to develope the data acquisition and control software.
Data analysis	We used MATLAB (R2019a, R2020b, R2021b) to reconstruct the TRIPS-OCT images from the photodetector readout. We used MATLAB R2021b to segment the TRIPS-OCT images, quantify the measurements, and conduct the statistical analysis. The central algorithm to reconstruct TRIPS-OCT images from triple measured Stokes vectors can be found at https://github.com/DrXinyu/TRIPS-OCT.

For manuscripts utilizing custom algorithms or software that are central to the research but not yet described in published literature, software must be made available to editors and reviewers. We strongly encourage code deposition in a community repository (e.g. GitHub). See the Nature Portfolio guidelines for submitting code & software for further information.

Data

Policy information about availability of data

All manuscripts must include a data availability statement. This statement should provide the following information, where applicable:

- Accession codes, unique identifiers, or web links for publicly available datasets
- A description of any restrictions on data availability
- For clinical datasets or third party data, please ensure that the statement adheres to our policy

Processed animal data (shown in Figs. 3 and 4), including en-face images and refractive errors, are available from figshare at https://doi.org/10.6084/m9.figshare.21300576. Additionally, one example guinea pig B-scan modulated by triple polarization states (shown in Fig. 1) is also available from figshare. The entire raw dataset of animal experiments is more than 25 TB in size and can be shared on request with appropriate data-transfer methods. For the clinical study, the raw data acquired during the study are available for at least 5 years from the corresponding author on reasonable request, subject to approval from the SingHealth Centralised Institutional Review Board. A request will be processed within 3 months.

Field-specific reporting

Please select the one below that is the best fit for your research. If you are not sure, read the appropriate sections before making your selection.

☒ Life sciences ☐ Behavioural & social sciences ☐ Ecological, evolutionary & environmental sciences

For a reference copy of the document with all sections, see nature.com/documents/nr-reporting-summary-flat.pdf

Life sciences study design

All studies must disclose on these points even when the disclosure is negative.

Sample size	As this study of PSB was a pilot study, no sample-size calculation was performed for the animal experiments, owing to the lack of previous studies. Empirically we decided to use 21 guinea pigs to investigate the correlation between refractive error and scleral birefringence. In the clinical study (Figs. 5 and 6), the sample-size calculation was based on the evaluation of the correlation between refractive error and TRIPS-OCT measurements with a 90% statistical power using preliminary parameters from the longitudinal guinea-pig study. For other experiments aiming for validation of the technology of TRIPS-OCT, biological independence is not required. Therefore, one animal was used for each specific aim.
Data exclusions	In the longitudinal guinea-pig study (Figs. 3 and 4), no data were excluded. In the clinical study (Figs. 5 and 6), we excluded 75 eyes (47%) with suboptimal positioning (50 eyes, 31%) and insufficient signal-to-noise ratio (average scleral SNR < 4.6 dB) from the sclera (25 eyes, 16%). The exclusion of images with insufficient intensity signal was pre-established. The threshold 4.6 dB was determined after the study because no preliminary TRIPS-OCT data were available. Perfect positioning of the scan was not always guaranteed in the clinical study because the positioning of TRIPS-OCT imaging head was not always accurate, owing to the lack of pupil alignment camera, fundus camera, eye tracker, or automatic depth-positioning unit. A sufficient signal from the sclera was not always guaranteed in the clinical study because a thick choroid may limit the light penetration to the sclera.
Replication	For the TRIPS-OCT technology, at least 294 volume scans (42 eyes, 7 weeks) were performed in the guinea-pig eyes in the longitudinal animal-model study; 180 volume scans were performed in human eyes, from which 100 volume scans were rated as high-quality, defined by the quality criteria in the human study. For the analysis of the correlation between scleral birefringence and the degree of myopia, no replication was performed because all validated data are included to achieve maximum statistical power.
Randomization	No randomization was applied because the study was focused on the correlation between TRIPS-OCT measurements and the degree of myopia.
Blinding	For the animal study, blinding was not possible because there was no prior knowledge of the association or correlation between refractive error and scleral birefringence. In the human study, The investigators were blinded to the birefringence measurements when segmenting the images, and were blinded to myopia status of the subjects during the processing of the TRIPS-OCT measurements.

Reporting for specific materials, systems and methods

We require information from authors about some types of materials, experimental systems and methods used in many studies. Here, indicate whether each material, system or method listed is relevant to your study. If you are not sure if a list item applies to your research, read the appropriate section before selecting a response.

Materials & experimental systems

n/a	Involved in the study	
☒	☐	Antibodies
☒	☐	Eukaryotic cell lines
☒	☐	Palaeontology and archaeology
☐	☒	Animals and other organisms
☐	☒	Human research participants
☐	☒	Clinical data
☒	☐	Dual use research of concern

Methods

n/a	Involved in the study	
☒	☐	ChIP-seq
☒	☐	Flow cytometry
☒	☐	MRI-based neuroimaging

Animals and other organisms

Policy information about studies involving animals; ARRIVE guidelines recommended for reporting animal research

Laboratory animals	A 1-year-old pig (Yorkshire-Landrace cross, Male, National Large Animal Research Facility, Singapore) was euthanized for TRIPS-OCT and PLM imaging. Three guinea pigs (Elm Hill Labs, Male n = 3, Pigmented n = 1, Chelmsford, US) aged 1, 12, and 16 weeks were euthanized for TEM histology analysis. 21 guinea pigs (Elm Hill Labs, Albino n = 17, Female n = 13, Chelmsford, US) were bred on-site for refraction-development analysis. 2 guinea pigs (Elm Hill Labs, Albino n = 2, Female n = 1, Chelmsford, US) aged 1.6 and 2.1 years were selected for TRIPS-OCT imaging from a group of breeders in our animal facility.

Wild animals	The study did not involve wild animals.
Field-collected samples	The study did not involve samples collected from the field.
Ethics oversight	The use of animals for these studies was approved by the Institutional Animal Care and Use Committee of SingHealth (AAALAC Accredited; 2018/SHS/1441, IACUC 1290).

Note that full information on the approval of the study protocol must also be provided in the manuscript.

Human research participants

Policy information about studies involving human research participants

Population characteristics	The population characteristics of each eye group used in the analysis are described in Supplementary Table 1.
Recruitment	80 normal adults aged 21 years and above without any known ocular diseases were recruited. The inclusion criteria were as follows: age 21 years and above; no diabetes and free from clinically relevant eye disease that interferes with the aim of the study, including glaucoma, diabetic retinopathy, age-related macular degeneration, uveitis, or vascular occlusive diseases. 10 patients diagnosed with pathologic myopia with staphyloma were recruited. For patients with pathologic myopia, the inclusion criteria were as follows: age > 21 years; both eyes presented staphyloma under wide-field OCT imaging. The exclusion criteria were as follows: eye conditions that may potentially result in poor quality imaging scans (severe cataract, corneal haze/opacity).<br><br>Normal adults were recruited from patients, visitors and staff of the Singapore National Eye Center. Patients with pathologic myopia were referred by clinicians of the Singapore National Eye Center. As a pilot study conducted in Singapore, 91% of the participants were Asian. Participants were compensated with 30 Singapore dollars each.
Ethics oversight	All procedures performed were in adherence with the ethical standards of the SingHealth Centralized Institutional Review Board (CIRB Ref No. 2021/2592). Written informed consent was obtained from all participants in accordance with the Declaration of Helsinki.

Note that full information on the approval of the study protocol must also be provided in the manuscript.

Clinical data

Policy information about clinical studies

All manuscripts should comply with the ICMJE guidelines for publication of clinical research and a completed CONSORT checklist must be included with all submissions.

Clinical trial registration	CIRB Ref No. 2021/2592. There is no public registration because the clinical study was not interventional.
Study protocol	Polarization Sensitive Optical Coherence Tomography — Phase II: A Pilot Study (R1819/61/2021).
Data collection	The data of patients and healthy individuals were collected between 15/02/2021 and 28/05/2021 from the research clinic in the Singapore Eye Research Institute. Healthy individuals were recruited from patients, hospital staff and visitors. Patients with pathologic myopia were recruited from general clinics in the Singapore National Eye Center and refereed by clinicians. Auto-refraction, axial length, age, sex and TRIPS-OCT data were extracted from the data-management unit of the institute.
Outcomes	Outcomes are not relevant because the study was not interventional.
